# Supplementary material for: Digital Forms of Commensality in the 21st Century: A Scoping Review
Source: Int J Environ Res Public Health. 2022 Dec 13;19(24):16734. doi: 10.3390/ijerph192416734 (PMC9779162; doi:10.3390/ijerph192416734)
Supplement: Supplementary file 1 [file ijerph-19-16734-s001.zip › Supplementary_Table_S2.pdf]

Description board of studies characteristics (n = 104)

Title	Year of Publication	Author (s)	Type of Study	Objectives	Main Results	Forms of commensality represented
Fast Food and Fast Games: An ethnographic exploration of food consumption complexity among the videogames subculture	2011	James M. Cronin and Mary B. McCarthy	Qualitative	To understand how food is used to create identity and community for gamers during core rituals. These meanings are to be explored within the broader context of subcultural experience in an investigation of the motives and the self-concept dynamics underlying this symbolic consumer behavior.	Food fosters feelings of solidarity, connection, belonging and devotion among players. It fulfills an important symbolic and identity role within the gamer subculture. Food sharing was typically observed during "social gameplay" rituals. The act of eating large amounts of junk food with the other contributes to the reduction of the feeling of guilt about the practice, as it is more socially acceptable, and also contributes to a type of rebellious behavior that denies the parents' and typically current ways of eating. outside the video game subculture. Still, there are exclusion, inclusion, rules and norms that govern the choice and consumption of food during events.	Young people practicing commensality while and after playing video games and interacting over food on video game websites and forums.
Cooking together	2012	Jeni Paay, Jesper Kjeldskov, Mikael B. Skov, Kenton O'Hara	Qualitative	Evaluate best practices for remote food preparation experiences to design digitally-enabled kitchens	The article showed how the spatial configuration of social interactions in the kitchen can be a relevant starting point for considering which and how technologies could support the act of cooking together with remotely distant family and friends.	Online videos of two people cooking together in residential kitchens
Eating alone, together: New forms of	2012	Catherine Grevet,	Qualitative	To Explore how technology might support altogether new routines and behaviors and	1) awareness: without any positive action, participants were able to become aware (be informed) about their friends. Such information was available all the time,	Friends informed each other about their dinners via an always-on-display app

commensality		Anthony Tang, Elizabeth Mynatt		mealtime activities for individuals lacking the setting of traditional commensality.	being part of the environment and social context of the dinner. However, such effects can only be used to the fullest if meals are taken in the same place as the Tablet (in the study, set in the kitchen).<br>2) Sociability: simultaneity (such as eating while being told that your friends are too, imagining that the other is also alone and probably in a similar room) generated a sense of company in the participants. Still, the act of changing status was referred to as a way of remembering others while carrying out their actions.<br>3) Interaction catalyst: knowing the status of the other, sometimes, was the starting point for other interactions, by other means of communication: "where did you go to eat?"; "what recipe are you eating?"	
Food for Talk: Phototalk in the Context of Sharing a Meal	2012	Kenton O'Hara, John Helmes, Abigail Sellen, Richard Harper, Martijn ten Bhömer e Elise van den Hoven	Qualitative	To explore how photographic materials and personal memory narratives could be sympathetically invoked within the broader social concerns of commensality, by using the 4 Photos System (a device that features autobiographical photos during meals)	The photo display is not merely a curiosity about the participants' past or a tactic to provoke small talk. It is a means of exchanging information of high personal significance and, therefore, an important factor in the construction (in the present tense) of relationships between diners. The photos, in a given situation, allowed a greater inclusion of a more "quiet and insecure" diner, in a comfortable way and without major constraints: "whose photo is this?". The photos served to bring up conversations about situations that diners eventually lived together or followed simultaneously because of common interests or friends. The photos contributed to the role of developing and strengthening family and affective bonds. The photos served for social and personal reflections on the discrepancies between past and present. The situations that demand a more sensitive management, so as not to embarrass/offend/exclude anyone, were circumvented with previously	Diners gathered around a table and interacted with each other as they interacted with their photos displayed by the 4 Photo Device.

					established rules or with the conversational dynamics of the meal itself. Preparing photos for dinner became one of the possible ways for diners to integrate and symbolically act in favor of sharing the meal, strengthening commensality.	
Telematic dinner party: designing for togetherness through play and performance	2012	Pollie Barden, Rob Comber, David Green, Daniel Jackson, Cassim Ladha, Tom Bartindale, Nick Bryan-Kinns, Tony Stockman e Patrick Olivier	Qualitative	Use the dinner party as a platform to explore how technology may support remotely located guests in sharing a dining experience together.	Adults to use a mobile phone at meals than for children	Diners shared the table remotely through the use of technologies that captured and projected their images on the tables of their respective geographically distant groups.
F-formations in cooking together: A digital ethnography using youtube	2013	Jeni Paay, Jesper Kjeldskov, Mikael B. Skov, Kenton O'Hara	Qualitative	With a view to creating interactive designs, investigate how people organize themselves spatially at home to cook together (cooking together) and share footage of these moments, based on the analysis of videos themselves and	The kitchen is a place of freedom and restrictions: there are several ways to get ready in the kitchen to produce videos of culinary preparations made with someone else. Such variety, however, collides with the spatial limitations placed by the kitchen itself, since it is constituted as a space filled by structures with little mobility. More than that, the arrangement of counters and appliances usually guides the positioning of participants in the scenario. The importance of	Participants in the videos prepare food together and share their experiences in communities on Youtube.

				Youtube communities.	positioning the participants for the fulfillment of roles and the organization of collaborative tasks during the preparations: people can be involved in the same task, eg. e.g. side by side or face to face, working in parallel in different stages or preparations, e.g. eg in a Z shape, one may be guiding or supervising the other, e.g. eg over the shoulder, just keeping company or acting to entertain the video audience, e.g. eg side by side.<br>Design: the recognition of such varieties supports the authors' hypothesis that the traditional placement of a single static camera that captures the entire environment does not account for all the actions and experience that can be filmed during this type of practice. In this way, they point out that the use of more cameras, adjusted to the chosen spatial conformations, can achieve a more intimate and informative dimension of the moment of preparations. Nevertheless, the strategic placement of microphones would fulfill a similar role.	
Food Media: Food and Technology as a Medium for Social Communicatio n	2013	Wei Jun	Mixed methods	Explore how media technology can be incorporated to utilize the power of symbolic and interactive activities around food, and the multi-sensory quality of food as a social medium, to enrich the way people interact through food and further enhance the mediated communication, exploring food messaging service enabled by food printing.	1) Around 80% of study participants indicate that the CoDine System is able to: 1) create a feeling of physically eating together.<br>2) An interaction between feelings is enhanced, when compared to a "purely digital" connection, compared to videoconferencing.<br>3) Together from the use of physical objects, sensitivity must be being compared. However, only 50-60% of participants agreed that the device was able to trace elements of their native cultures, and that meals using CoDine were "normal" (non-strange).	Remotely connected diners use the CoDine System as a videoconferencing device that also allows for personalization and interaction through digital tablecloths and also the sending of written messages through physical food printed by a 3D printer.
Consuming	2014	Pepukay	Qualitative	To explore the impact of	Family identity from multitasking: all families had the	Participants watch TV while

family quality time: the role of technological devices at mealtimes		i Chitakunye e Amandeep Takhar		electronic device use on family meal rituals	habit of using the TV during meals, so the authors concluded that such behavior reflects not only the experience of each family, but also points out that it is already part of "a collective sense of family identity". A call from a family member who was away from the family dinner was observed: the cell phone was used by more than one family member, and worked as a form of maintaining the connection between present and distant family members and also organizing the family routine.<br>Still, in a family where the father was always away for dinner, facetime was used to include him in the conversation. The same happened with friends and cousins. This practice was understood as a positive modernization of meals that traditionally depended on the physical presence of all participants.<br>Individual identity from multitasking: the use of technologies to reaffirm roles, identity and identity. One participant reported using his cell phone during dinner to chat and then shared the private conversations with his family. Tensions: Conflicts have also been reported due to the use of devices at the table, to the detriment of interaction with those present.	eating, use phones to make calls and video conferences while at the table, and also use cell phones to discuss their routine during meals.
Eating online discourses: rhetorics consumption on contemporary Bicocca (Milan, Italy)	2014	Luca Rimoldi	Qualitative	To reflect on the rhetoric of food consumption in two historical tractors in the city of Bicocca, Italy, based on comments on restaurant review sites and on the tractors' own websites and blogs.	Aldo's Tuscan trattoria was once frequented by industrial workers. But, since industrial activities were shut down in the neighborhood, it has been frequented largely by university students, due to the geographical proximity between Trattoria and the University of Milan. This process can be observed in online reports, in which students appreciate the trattoria's historic (and rare) decor and atmosphere, inherited from its past as a working clientele. On the other hand, the owner of the trattoria also recognizes this movement	Diners build and share meaning and identity about their experiences in trattorias

					in his online posts and, recognizing the need to adapt to the university profile, offers, for example, the exchange of meals for small tasks in the tractor. Still, there is a series of posts associating trattoria with communism, placed by the author as a political and historical commensal expression, which locates commensals from symbols attributed to food from the intersections between labor movements and the way university students relate to each other. with that past. Trattoria Arlati, on the other hand, is observed in the online discourse as a place that brings together layers of different eras experienced by the Bicocca neighborhood in its decoration, so that Trattoria Arlati worked to increasingly integrate itself into the Milanese urban circuit of street establishments, as its own website suggests: "over the years, the city has changed", while not giving up its traditional past. Thus, Trattoria Arlati is built on a discourse of generational encounter. Likewise, the commensality of the place is based on remembrance, on the celebration of a Milanese identity.	
Fridgematch: Design Probe Into The Future Of Urban Food Commensality	2014	Denisa Kera e Nur Liyana Sulaiman	Qualitative	To investigate how new forms of commensality can reduce the negative effects of "(food) alienation" and fast food culture by creating new types of economic chains and social networks around food.	"Proved" a possibility of future forms of food commensality based on interaction between strangers via social networks and application and the emergence of alternative family and friendship structures in cities.	Users of an application indicated which leftovers/leftover ingredients were in their refrigerators and, later, arranged meetings to cook and eat new dishes from these foods.
Pairing technology and meals. A context enquiry in the	2015	Hasan Shahid Ferdous, Bernd Ploderer,	Qualitative	To understand how different factors impact our technology choice during mealtimes and how we often make conscious (and	Technologies are linked to meals, however, the inverse was not observed: diners do watch the masterchef while they eat, but they would not change the meal times if the show started to air later. Except for specific occasions when a greater degree of	Diners interact with each other using TV programs, streaming, Youtube, music, applications that control the lighting of the environment.

family household		Hilary Davis, Frank Vetere, Kenton O'Hara		unconscious) choices in this regard.	formality or a greater concern regarding the interaction between diners is cultivated, the rules for the use of technologies at the table seem to be malleable and adapt according to the situation of each meal. They can be used to encourage the interaction of diners in a positive way (when they get together often to watch a TV show together or to give a themed air to the meal on account of a birthday, using music and youtube on TV). In cases where the food itself is special or very dear to diners, it starts to receive more attention and the use of technologies tends to decrease.	Furthermore, forms of "remote commensality" are mentioned in the theoretical formulation of the article.
Technology at Mealtime: Beyond the "Ordinary"	2015	Hasan Sharid Ferdous	Qualitative	To explore which communication technologies generally are used and shared during the family mealtime and how they are used	The analyzed data point to occasions when technologies leave their peripheral place and assume an important role: from unwanted to desired, from ordinary to tools of celebration. 1) Spatial arrangement: it was observed that certain technologies were not only placed close to the meal places, but that the family was also distributed so that everyone could have access to it. Still, there were "hidden" technologies, but available if required.<br>2) Mutual Modeling of Technology and Commensality: Technology often served as an agenda for conversations. There were also situations in which she was in the background, receiving attention at specific moments. Technology has proved to be a routine element of family meals, although this is not due to a specific type of technology, but to a certain emerging type of promoting meals.<br>3) Celebration: on special occasions, technology was used to create different scenarios/environments than usual. The type of technology used in the routine and on the special occasion was the same, only the creativity when using it was different. 4) Distraction: considered, in other studies, as one of the damages	Families use in-house technologies to spark conversations during meals or to create a certain effect on the environment on special occasions.

					caused by the increase in the use of technologies during meals, here, it was understood as a desired effect on certain occasions (making children finish their dishes or preserving harmony" the table").	
Commensality and the Social Use of Technology during Family Mealtime	2016	Hasan Shahid Ferdous, Bernd Ploderer, Hilary Davis, Frank Vetere E Kenton O'hara	Qualitative	To present the contemporary role of technologies in family meals, exploring how certain characteristics of digital technologies affect the spatial configuration of these meals (observing positive and negative aspects for commensality and also how to deal with the negatives)	Television proved to be a well accepted and normalized component of interactions at the table, while other technologies were, in general, left out or caused strangeness and drew attention when they were present (e.g.: phone ringing). Ready-to-hand technologies (indistinguishable extensions of the participants' action/interaction): televisions or other screens for streaming, smartphones (with restrictions, mainly for children). Present-at-hand technologies (available for quick access): video games, laptops (used before and just after dinner, but not during, remaining connected to the table). It was also possible to observe a situation in which technology guided families (e.g.: sitting around the TV) and vice versa, when families guided technology (eg, taking devices and bringing TVs closer to the table). On other occasions, devices remained close but hidden, so that they were at hand if necessary, and, finally, devices that were deliberately taken away from the place of meals (associated with rules for homes with children). Regarding the use, technology could be observed as a mediator of interactions, as a way of coordinating the logistics and tasks agreed between family members, as the holder of attention (TVs with very high volume), use of technology to distract and, still, behaviors in the sense of considering the interaction and use of technology, and use of technology on special occasions (creating a period environment with a Youtube video, for example).	Family members used different ways of socializing from TVs, apple TVs, dvd players, smartphones, notebooks and other devices of the represented houses

Pop-ups, meetups and supper clubs: an exploration into online mediated commensality and its role and significance within contemporary hospitality provision	2016	Gavin John Urie	Qualitative	To explore the role and importance of Online Mediated Commensality and its place in the provision of contemporary hospitality	Control: control before the meal: all events were shared through digital media, providing basic information about the organization and the type of experience offered. All dealt with the style, date, time and place, although the precise location was sometimes only made available to those who confirmed their participation and as the event approached. Also, details about the menu, such as the offer of alcohol, were commonly communicated. Likewise, the hosts commonly showed interest and concern about possible dietary restrictions of their guests, which was also observed in the event announcements. Such behavior by the hosts sometimes served to exclude participants, sometimes to make their inclusion viable. The author, immersed in the events, noticed that, routinely, people received adapted dishes, which could only have been arranged before the meal. Usually, the events already came with a defined end time. Informal control of the organization: in addition to the information previously agreed, situations were also observed in which the organization took place in an informal way, throughout the events: sometimes, envelopes were left on the table with the payment of the meal, sealing a tacit agreement between guest and host that, until then, did not exist. Likewise, when the events took place in houses, it was implicit to the diners which rooms were available for circulation and which were restricted, without it being necessary to verbally express such information. Gender roles: Men competed for the best stories and commonly occupied the head of the tables and initiated conversations. Still, situations were observed in which women contested this configuration and assumed the leadership and head of the tables. Nevertheless, according to the author, women were	Conventional dinner gatherings organized digitally

					seen (including in events that sought leadership) occupying more "maternal" spaces, making themselves available to wash the dishes, for example.	
Social Eating: una ricerca qualitativa su motivazioni, esperienze e valutazioni degli utenti della Piattaforma VizEat.com [Social Eating: A Qualitative Research on the Motivations, Experiences and Ratings of Users of the VizEat.com platform]	2016	Grazia Bastasin	Qualitative	To analyze the phenomenon of social eating (eating with strangers), focusing on the dynamics of users of the social food platform VizEat, in order to understand the reasons that lead participants to eat with strangers and whether this practice can become a source of income for the hosts.	Reason for participating: pleasure in sharing moments, desire to meet new people and get to know perspectives on life and new ideas. Cultural reason: desire to know the culture and history of the other diners (interest not only in the food, but in the other). Economic reason: the hosts seemed to focus more on the experience than on the profit, although the guests saw this dimension as a negative point, since the prices were on par with the price of restaurants (they found the experience expensive). Flexibility, accessibility and trust: it can have a favorable or unfavorable effect on the motivation of the participants: the dinner options can be defined only by the host, or he can consult his guests. The number of guests can be established in advance, which, in some cases, could represent an obstacle. Still, there are trust issues to consider: “Do I go to a stranger's house? Is the kitchen clean? What comments and evaluations did the stakeholder profiles receive?” Eating and cooking together (the experience itself): sharing the meal: the home environment decreases surveillance over behavior (relaxation). The act of eating together is understood as a space for exchange and mutual knowledge → the very act of eating with the other is a full movement of encounter between subjects. Cooking with others: "in the interviewees' perception, cooking is an act of love, commitment and gift. It is a moment of meeting, learning and fun. Cooking with other people involves communication, negotiation, attention and respect for objects, rules and people". Perfect dinner: feel at home, clean environment and	the host organized the event and posted online on social platforms created, while the guest, after registering on the same platform, chose the event to attend, paid the amount requested by the host, and went to the host's house at the set time to have a "food experience"

					good food. Habit and Food Decisions: The way participants choose the menu can move you away from or closer to an agreement about the event. The social eater: the participants, when imagining the profile of their peers, described people with an open mind, courage and positive personalities.	
Synchronized Dining Tangible mediated communication for remote commensality	2016	Judit Komaromi Haque	Qualitative	Discuss commensality as an important social act, as well as examine the role of touch as a channel of communication. Finally, the work sought to present a way to "unite" couples during meals held remotely, using interactive devices.	Prototypes created with remote commensality in mind: 1) Cuddly: a device that creates a sense of togetherness between couples who are practicing remote commensality. It has sensors that, when stimulated by the user's touch, produce tactile feedback and massage your hand lightly. (Each one uses theirs during the meal). 2) Dinner Companion: a device attached to the forearm of couples who are practicing remote commensality. The device vibrates in response to minimal movements of the user's arm, in addition to producing heat, producing the sensation/experience of union between geographically separated diners. → "This thesis project introduced a new approach for using haptic feedback technology, for enabling interpersonal communication. Thus encouraging to explore new possibilities for haptic interactions. However, the design process is incomplete, requiring several additional stages, for understanding and fine tuning the final designs. More work, to additionally finetuning the design and conducting user testing, would be necessary steps".	Diners used technologies that provide tactile interactions as communication channels in remote meals
TableTalk: Integrating Personal Devices and	2016	Hasan Shahid Ferdous, Bernd	Qualitative	To explore the relationship between technology and our everyday commensality presenting TableTalk, an	1) The TableTalk device did not interfere with the use of typical tableware (glasses, plates, etc.). All were able to fully access the screens due to the use of turntables (lazy susan). TableTalk didn't disrupt the	Diners used TableTalk to interact over the contents of their personal devices (Iphones and Ipads) while

Content for Commensal Experiences at the Family Dinner Table		Ploderer, Hilary Davis, Frank Vetere, Kenton O'Hara, Geremy Farr-Wharton, Rob Comber		application that integrates the personal devices of mealtime guests into a single, enabling the sharing of personal content (e.g., photos, music, social media posts) for the collaborative construction of a mealtime experience. Also, understand how different design aspects of TableTalk impacted commensal experience and the evolving behaviors around this device ecology; and, for last, offer new opportunities for design and novel understandings of the potential role of technology for commensality in the family home.	normal flow of conversations: most of the time, attention was focused on family members and not the device.<br>2) The pairing of personal devices faced mishaps, such as: technical incompatibilities, absence of family members or poor adherence to smartphones. However, it was observed that sharing content was already common among family members, demonstrating the complexity of interactions between them based on technology: there were tablets for shared use with files of several members of the household and even personal use tablets were possible. find photos and music from other family members. Such collaborative and sharing attitudes could also be observed at the table using TableTalk.<br>3) Moderation of the content that would be shared was more frequent in homes with children. Adults, in general, only bothered to remove content that they thought would not be interesting for the rest of the family. Parents are also concerned about ensuring that their children have space to show their files, avoiding feelings of exclusion. Teens shared fewer files about their personal life.<br>4) Third-party tweets and photos were the most shared content.<br>5) TableTalk has successfully sparked conversations between family members. They showed interest in why their relatives chose certain content.<br>6) Although allowed, families do not use technologies other than TableTalk during meals.<br>7) TableTalk was not the heart of the meals. The everyday conversations and beyond what was shown by the device remained throughout the meals, putting TableTalk in the position of an ambient technology that sometimes took a back seat. When songs were	dining.

					played, the device tended to garner more attention from those present, whether to provide amusement or irritation. Finally, Tabletalk made family members stay longer at the table after finishing their dishes.	
Technology at the Table: Attitudes about Mobile Phone Use at Mealtimes	2016	Carol Moser, Sarita Schoenebeck, Katharina Reinecke	Quantitative	To explore attitudes about mobile phone use during mealtimes and consider the design of mobile technologies for supporting mealtime behaviors. To do so, we investigate the following two research questions:<br>1. What are people's attitudes about mobile phone use during mealtimes?<br>2. What factors contribute to people's attitudes about appropriate mobile phone use during mealtimes?	Results show that participants find it more appropriate for adults to use a mobile phone at meals than for children.<br>Participants rated adults texting and adults answering a call as more appropriate than inappropriate. On the other hand, children texting and both adults and children going online or using social media were rated as more inappropriate than appropriate<br><br>Quanto mais a pessoa usa o celular durante as refeições, mais ela acha adequado que outros também o façam.	Commensal situations with cell phone use
Celebratory Technology to Orchestrate the Sharing of Devices and Stories during Family Mealtimes	2017	Hasan Shahid Ferdous, Frank Vetere, Hilary Davis, Bernd Ploderer, Kenton O'Hara, Rob Comber, Geremy	Qualitative	To understand how celebratory technologies change/affect family interactions during meals.	1) The influence of chorus on dinner preparation: the app made family members think better about what they would have for dinner. At dinners without it, it was common to effortlessly choose a TV program for dinner, while with it, the act of sharing some content was more elaborate, personalized and democratic (everyone had their turn to choose what others would see). 2) behavior in relation to technology: in traditional meals, the individual use of technologies could cause discomfort in the other dinner participants or be unwanted by parents, while in dinners with the application, its inclusive use provided positive interactions between family members. 3) The application was used to update the family about news	Family members used the Chorus application to share content previously chosen by each of them while they had dinner.

		Farr-Wharton			and moments when members were not together; to remember shared experiences between them; and to reflect on and remember certain subjects. 4) changes in conversations: when watching TV, the families hardly talked and looked at each other, while at dinners with Chorus, they interacted more and paid more attention to each other. At the same time, they also paid more attention and talked better about the food itself.	
Digital food and foodways: How online food practices and narratives shape the Italian diaspora in London	2017	Sara Marino	Qualitative	To exam transnational practices of mixing and matching italian and britannic culture involving not just buying and consuming food, but also taking into account a myriad of other actions including buying the right ingredients, sharing a meal with other members of the same diaspora and adapting recipes and conversations about food. Also, the research brings another element to the relationship between food and migration, which is its online reconfiguration	Virtual commensality: in the interviews, it was observed that part of the Italian immigrants in London practiced virtual commensality (zoom videoconferencing). The practice was used as a form of exercising Italian food culture. Virtual commensality increases the sense of identity and "normality", bringing them closer to Italian food rituals and to a new family conformation that is able to include those who are far away.<br>Virtual commensality demanded adaptations from both sides (time zone). The creation of visceral food spaces: feeling at home, feeling homesick and establishing a connection between the old house and the new house, in a type of construction of a transnational domestic space (with culture, co-presences, "smells virtual environments", familiarization with the environments). Sense of collectivity and identity: food and online communities also served to strengthen the affirmation of immigrants' identity, as well as in the identification of their peers and in the practical expression of their sociability. Participation in communities also encouraged commensal practices in their country of origin, complementing the practice of virtual commensality (the dissemination and sharing of	Video calls between family members residing in Italy and Italian immigrants in London. As well as sharing Italian foodways (recipes, expressions, photos...) in online communities.

					legitimately Italian recipes and dishes. Likewise, Italian foods fulfill a role of social capital and reinforce ties among community members.	
Food porn, pro-anorexia and the viscerality of virtual affect: Exploring eating in cyberspace	2017	Anna Lavis	Qualitative	To explore food porn and pro-anorexia communities, from the lens of the role of the act of eating in the production and mediation of individuals, everyday lives and spaces, so that one can think about a "food/food biopolitics", both in a visceral an affective perspective.	Food Porn (FP) is related to corporeality, extrapolating the notion of materiality. From the stimulus and "feeding" of "different hungers" and desires, FP incites affections, imaginaries and cultural manifestations. FP enables a "consumption of the other" in posts that refer to some kind of past experience or in scenes that go back to the practice of commensality (nostalgic appeal): food porn points to a past act of eating and/or serves as trigger for a future. The use of food porn as a weight loss tool not only operates by preventing the materialization of the offline eating moment, but also by serving as an element of satiety. It is a form of ingestion without being incorporated, in a way that consuming virtual food is also eating.	Participants in pro-anorexia communities share ("eat") food porn images and pages with each other ("with others").
Food porn' or intimate sociality: committed celebrity and cultural performances of overeating in meokbang	2017	Glen Donnar	Essay	To analyze how mukbang performances engage with the economic and cultural tensions of the DIY celebrity wave	Mukbang is a form of food porn, understood as something that its public can't do, but enjoy watching. The mukbang is a form of expression of DIY culture (an important social space for the emergence of celebrities who use new technologies to simulate intimacy and generate affection).<br>Eating is a continuation of oneself and one's corporeality: eating can be a mundane exposure of the visceral nature of our connection with others, with ourselves and with the environment."<br><br>By eating, female BJs produce, reproduce, resist and transform gender ideologies.	Mukbang - a person eating large amounts of food while streaming.
Novas	2017	Maria	Narrative	To reflect on the changes in	↑Eating alone and eating on the street. The social and	Meal sharing across

configurações do comer junto – Reflexões sobre a comensalidade contemporânea na cidade de São Paulo (Brasil) [New configurations of eating together— Reflections on contemporary commensality in the city of São Paulo (Brazil)]		Henriqueta Gimenes - Minasse	review article	the configurations of eating together in the last 50 years, starting from a macroscopic panorama of a globalized world to the city of São Paulo.	affective element is still important for Brazilians.<br>Creation of numerous blogs and online communities dedicated to food. ↑ Food apps and food courts.<br>↑ exploration of gray areas between public and private in the construction of new ventures → "feel at home".<br>Relativization of proximity made possible by advances in communication technologies.<br>Diversification of ways of interacting with others:<br>Meal sharing across websites and apps (Crushing Table and EatWith);<br>Kitchennd: uniting cooks and home-cooked foodies;<br>Meal Sharing (paid home-cooked meals: experiences between diners and cooks);<br>Eat Feastly "a platform that promises to unite chefs, amateur cooks and diners around the world".<br>Also, the increase in the use of smartphones and gadgets for making calls and videoconferences to meet with each other remotely during meals.<br>There is also the possibility that technologies disfavor commensality, as when it creates tensions and distances due to cell phone use during meals.	websites and apps (Crushing Table and EatWith). Kitchennd and Eat Feastly: uniting cooks and home-cooked food lovers. Meal Sharing (paid home-cooked meals): experiences between diners and cooks). Also, creating blogs and online communities around eating and using smartphones and gadgets to make calls and videoconferences to meet with each other remotely during meals.
"Table manners": Children's use of mobile technologies in family-friendly restaurants	2017	Hilary Davis e Hasan Shahid Ferdous	Qualitative	To exame the mobile technology usage by children during mealtimes to understand what it is that children are doing with the technology, how technology is balanced alongside eating a meal, and how these activities are mediated and managed by others to ensure a positive dining experience.	Table setup and technology introduction: In all videos, children asked permission to use electronic devices. In two cases, parents took the time to configure their own devices as routers to support their children's use of technologies. The purposes of the uses were varied: research, social networks, games.<br>In the other two cases, the children did not have access to the internet.<br>The children had laptops, iPods, cell phones, etc.). They all had their own smartphone.<br>In general, their devices were positioned in front of	Families eating in restaurants while their children were using electronic devices

					them, allowing other diners to also see the screen. Exceptionally, the devices could be on the lap (when the table was full). Also, parents warned their children about the danger of leaving the devices near jugs and drinking glasses.<br><br>Use of technology and meal timing<br><br>Children started using technology when the whole family sat down at the table. As snacks and drinks arrived, the children continued to use it. Discontinuation of use only occurred when main courses arrived, with exceptions (one child took longer to put away their device because they were saving their game, which was seen by parents as a reasonable reason to prolong use; in another case, one of the children insisted on using it even after the father asked her to stop using it).<br><br>Purpose of use: playing games, interacting on social networks, watching videos, learning, creating videos and mixed media use (combination of analog elements such as family games or use of napkins to draw + photographs, creation of posts, use of apps) .<br><br>There were also negative findings: children with headphones who interacted less with the family and competitive use of games and device sharing associated with table discussions.	
Technologies are coming over for dinner: do	2017	Alexandra Urbano Magalhã	Quantitative	To understand how parent and child digital technology use during mealtimes influenced children's	The results indicate a significant negative effect of parental participation in technology use on children's participation in the ritual. The result is not repeated when children use electronic devices.	Meals in families with children using electronic devices

ritual participation and meaning mediate effects on family life?		es Quaresma		participation in the ritual, considering families with preschool and school aged children.	Furthermore, the meaning of food ritual was associated with family cohesion, parental satisfaction and couple satisfaction, but not with parental self-efficacy.	
"Arm-A-Dine”: Towards Understanding the Design of Playful Embodied Eating Experiences	2018	Yash Dhanpal Mehta, Rohit Ashok Khot, Rakesh Patibanda, Florian ‘Floyd’ Mueller	Qualitative	Introduce a new form of prototype that sheds light on opportunities for using technology to support social eating. Show initial results from Arm-a-dine (robot arm). Finally, articulate themes to expand the understanding of the interactional HFI.	Arm-A-Dine facilitated engaging conversations around food and the way we eat besides facilitating incidental bodily movements and empathy towards the eating partners	Participants shared a meal using a robotic arm attached to their body.
“You Better Eat to Survive”: Exploring Cooperative Eating in Virtual Reality Games	2018	Peter Arnold, Rohit Ashok Khot, Florian 'Floyd' Mueller	Mixed methods	To investigate the use of a gustatory interface, in particular the chewing of food, to control actions in VR games AND to address the feeling of social disconnect in VR environments through cooperative eating, which could add excitement and the feeling of social presence to the VR experience.	Eating real food increased players’ feeling of presence, challenged trust dependencies and made the survival aspect of the game feel more “real”. Eating interaction helped to build trust	The player in the real world must feed the player in the virtual world. Without cooperation, the two lose the game.
(Dis)comfort food—Connecting food, social media, and first-year	2018	Michael Pennell	Pedagogic experience report	To reflect on the discomfort experienced in a pedagogical practice between teacher and students regarding tensions and limits related to food in social media.	Despite the pedagogical proposal being evidenced by the company of social networks and the comfort of food, the experience of discomfort and discomfort for the students. They felt free to post every day and when outsiders felt their space was invaded. students in their personal profiles, due to their professional	Sharing photos and food recipes on blogs and social media.

college undergraduates					relationship.	
Application of the critique of dispositives to the performative dinner "El Somni" by El Celler de Can Roca and Fran Aleu	2018	Anne-Claire Yemsi-Paillissé, Yanet Acosta Meneses, Monique Martínez e Elvira Calvo Gutiérrez	Qualitative	To apply the critique of dispositives developed in the laboratory LL@-reatis of Toulouse since 2000) for the first time to an experimental gastronomic project performed in Spain, called “El Somni” (“The Dream”).	Technical aspects: El Somni was set up in an art gallery in Barcelona, on a table with 12 seats. Unlike the trend, the kitchen was completely hidden. The table was equipped with 5 flat screens positioned just behind the diners and Roland Olbeter's robots played music. Cameras and projectors were placed on the ceiling.<br><br>Pragmatic aspects: the language and design of the dinner followed the "French style" of social behavior and rules. The table was round and the dishes were served simultaneously. The diners were from different areas: a chef, an anthropologist, a virologist, a plastic artist, an actress, a director... Also, the number 12 alludes to the Christian tradition: the 12 apostles and the Eucharist as an anthropophagic ritual. On the occasion, rare meat was served that represented the body of Adonis, while images of him were displayed on the flat screen.<br><br>The role of the canvases: canvases are a poem illustrated by music, dishes and wines. All images are interconnected with other interpretive elements of the meal. The prominence of screens alters the social dimension of food. The performance mediated by the large screens and lights changes the normal rhythm of conversation and ensures that the El Somni experience is much closer to a movie theater or theater. Even so, it was possible to isolate the typical gastronomic experience from commensality (referring to dialogues), both at pragmatic and symbolic levels.	12 guests ate 12 courses with 12 different wines in 12 acts (located at Arts Santa Monica, Barcelona).

					The symbolic/axiological dimension: a transgressive menu: by centrally approaching death in its artistic and literary dimension — and, in this case, gastronomic — the total experience breaks the typical expectation of gastronomic experiences, associated with happiness, and offers diners sensations primitives of "fear", and eventual displeasure, sadness, regret and similar emotions.	
At the intersection of thinness and overconsumption: the ambivalence of munching, crunching, and slurping on camera	2018	Astrid Schwegler-Castañer	Comment - Essay	To explore the cultural phenomenon of Mukbang and offer new insights into the ways that food, femininity and user-generated content intersect.	There was a normalization and acculturation of fit bodies, made up and submitted to aesthetic procedures in Korean culture, embedded in a moral critique of divergent bodies. sometimes associated with the fetishization of female bodies, sometimes as a liberating practice in the face of social expectations about bodies.	Mukbang broadcast by women shown on Youtube
Comensalidade.com—Uma reflexão introdutória sobre as novas tecnologias e as práticas do comer junto [Commensality.com—An introductory reflection on new technologies and practices of eating	2018	Maria Henriqueta Gimenes - Minasse, Pedro Henrique Drudi, Andrea Ortolani Faltin e Mariana da Silva Lopes	Review Article	To reflect on the use of technologies during the practice of eating together	There are new technologies and digital services that increase the possibilities and varieties of the practice of commensality — although their mere existence do not necessarily lead to that increase in the practice: such as services and blogs of information and tips about restaurants and delivery apps. In another branch of novelties, there are sites and applications whose purpose is to promote commensality, such as sites that unite people in favor of gastronomic experiences of a loving or professional nature; or even services structured from hosts (amateurs or professionals) of gastronomic experiences. Also, there are services aimed at guiding tourists in search of local food. From these findings, it is possible to conclude that the internet offers a creative space for a series of different practices, with the involvement of the market, diners	Digital dating services for dining experiences and online communities centered on dining experiences

together]					and chefs. Such novelties, according to the author, are made while commensality persists, with the possibility of relativizing physical presence and creating sharing flows by photo, video and other media. Nevertheless, the author also makes the counterpoint that there is a dimension of the use of these technologies that inhibits or hinders the commensal practice.	
Peligro en el plato: Rumores y leyendas urbanas del tema alimentario en internet [Danger on the plate: Rumors and urban legends of food on the internet]	2018	Oscar Eduardo Rueda Pimiento	Qualitative	(1) To analyze the relationship between food, culture and the internet. (2) To contextualize the role of fears, mediators and media on modern food and (3) analyze the potential of internet anonymity to trigger the narrative of personal stories	(2) Media and mediators interfere with our perception of fear and risk. With the instantaneity of the internet, the media platforms became creators of public emotions through limited texts in terms of all possible kinds of bias.<br>In this context, the creation of conspiracies, denunciations of unpleasant and harmful events associated with food form a body that can be defined as "food fraud".<br>The KFC case: images of a KFC snack with "shapes that allude to a mouse" quickly circulated on social media, in a possible update of previously told urban legends, such as the mouse in coca-cola. The repercussions motivated the investigation of the meats, which resulted in the confirmation that the snacks were indeed made with chicken meat. When analyzed, this case corroborates the argument that these scandals are profitable and scalable, especially when dealing with multinationals or Asian companies, in order to resonate and reinforce the fear felt by the population regarding the hygienic-sanitary conditions of food.<br>However, there are also cases of urban legends focused on food taboos involving traditional cuisine, such as the use of prohibited meats – for biodiversity protection – in traditional recipes. All these processes, according to the literature, point out that fear and	Food mobilization in favor of activism on the internet, in addition to social life around fears and rumors about food on social networks

					mistrust stem from the feeling of vulnerability in the face of market structures that are placed on societies and the laboratory production of food; contact with the exotic or extremely modern; and even as updates of past legends.<br>(3) The stories are forms of politicization, food activism and attempts to build alternatives for the homogenization of food cultures; and to act on the patrimonialization of food.	
Eating (alone) with Facebook: Digital natives' transition to college	2018	Giyoung Park	Mixed methods	This study aims to investigate college freshman students' communication patterns in daily social settings and to explore the relationships among their communication patterns, associated contextual variables, and adjustment in college.	Dormitory dining halls were among primary social settings on campus; yet, freshman students did not want to eat dinner alone there. Dinner location was not correlated with sense of belonging; however, underrepresented minority (URM) who had dinner in dormitory dining halls reported lower sense of belonging. URM solo-diners more likely used a screen such as a mobile phone. Solitary URM diners, especially those who used a screen, reported lower sense of belonging early in the initial semester. Text messaging in the dining halls was associated with a greater sense of belonging. Furthermore, greater sense of belonging was related to better mental health. URMs' greater sense of belonging in the initial semester could predict greater academic achievement over the next few semesters; however, non-URMs were unrelated to GPAs.	Dynamics among university students in the university cafeteria
Comida y Mundo Virtual: Internet, Redes Sociales y Representaciones Visuales	2018	José Antonio Vázquez-Medina e Francesca Bayre	Qualitative	To observe how the images of the facebook page "So Mexican" appeal to a sensorial memory for the construction of a specific community that, through vision, refers to taste, smell	1) The dichotomy (this is mexican/this is not mexican) is a highly present element in the posts on the page and acts to classify, include, exclude and validate practices among the members of that community. Furthermore, they build rules on how to identify and recognize Mexican foods and how to put into practice the sensory records of the past. 2) The dichotomy also	Participants on the Facebook page share images that refer to sensory experiences from their memory as Mexican immigrants.

				and touch, creating an articulated food experience	serves to manage and build stereotypes about Mexican eating. In this case, it plays an important role in the totemization of dishes and practices, in the expression of rites of passage and everyday elements. Despite the humorous character, they are aligned with the construction of symbols easily recognizable by community members.	
Mauvais à regarder, bon à penser: il food porn tra gusti e disgusti [Bad to look at, good to think about: Il food porn tra gusti e disgusti]	2018	Simona Stano	Mixed methods	To reflect on the "disgust" directed at food porn on social media	There is a denial of food pornography - a "non-food pornography" - which is a "degenerate form" of food pornography that, mocking it, ends up adopting its same forms and the same functional mechanisms in order to neutralize it. Pavlovian salivation effect with an equally strong disgust, extolling the "non-food", which cannot be eaten, either because it was poorly prepared or because of bad condition (rotten, dirty) or another factor inherent to the nature of the food or object shown.	People build a community around the sharing of "monster dishes", culinary failures, junk foods, in a counter-foodie movement.
TableTalk: Staging Intimacy Across Distance Through Shared Meals	2018	Ana-Christina Acosta Gaspar de Alba	Qualitative	To explore how commensality, or the shared meal, can be used to structure a series of scenes between two characters in different locations, using TableTalk, a play in development as an example of the potential for long distance commensality in theater	Commensality seems to be a valid path for the development of multi-localized theater plays. If the play gains international contours, it is worth thinking about adapting the dishes prepared and consumed by the characters. The use of real kitchen and food utensils would increase the audience's sensory experience. However, due to the theoretical and still-developing nature of the study, greater efforts will be needed (such as actually presenting the play to a real audience) so that further conclusions can be drawn.	The characters share a meal via videoconference, each on their respective stage, performing for their respective audience.
Technology at Family Mealtimes	2018	Hasan Sharid Ferdous	Qualitative	To examine the everyday interactions of familial uses of technology around mealtimes and investigate how family members	Technologies are integrated into the mealtime activities in a dynamic fashion and provide a balanced account of the affordances and challenges of technology usage during family mealtimes. This study reveals certain circumstances in which background	Eating while using digital devices, including TableTalk

				configure the dining space and the technologies within it. Understand how technologies are used and negotiated amongst family members and the influence of these technologies on the content and context of their interactions. Investigate how the everyday technologies can be used to enhance the familial experience of eating together and create new experiences for togetherness in this space.	technologies come to the foreground, visible devices are hidden, unwanted distractions become desired, and ordinary technologies support celebratory occasions. I identify four patterns of arranging technologies and family members around the dinner table and discuss how technologies contribute to satiety and commensality. The findings from this study also show that a technology does not require being special or separate from the ordinary technology used in everyday life to support the special occasions in the family, rather it achieves this feat through its usage in special ways.<br><br>Technology usage during mealtimes is traditionally viewed as impeding social interaction at family mealtimes. However, this research demonstrates how technologies can support, enhance, and augment social interactions, mediate usage, and resolve tension among the family members during mealtimes.	
The use of Māori and Pasifika knowledge within the everyday practice of commensality to enrich the learning experience	2018	Nan O'Sullivan e David Hakaraia	Essay/ pedagogic experience report	From commensality, to appreciate the reciprocity and respect instilled in Kaupapa Maori and in the ideology of the ta-vaan of the Pasifika culture (based on a particular notion of time and space) and to discuss how such themes can contribute to Design by being purposefully, consciously and respectfully immersed in the creation and praxis of designs.→ exemplify design solutions	The article brings Smart Treys (the result of other work) — an application guided by the Maori and Pacifika notions of the tangible and intangible that allows you to connect remotely during meals — to support that technologies are key to, combined with culture, and behavior, create sustainable solutions that promote and democratize the union (togetherness).	Diners connect via Smart Treys, an app guided by Maori and Pacifika notions of the tangible and intangible that allows them to connect remotely during meals

				taught by students to combine the traditional wisdom of the aforementioned peoples, social connection, cultural identity and creation of learning places.		
Toward new forms of meal sharing? Collective habits and personal diets.	2018	Estelle Masson, Sandrine Bubendorff e Christèle Fraïssé	Mixed methods	1) Place the rise of dietary practices into a general context in which the relationship to food tends to be individualised. 2) Evoke the specificities of the French relationship to food, and analyze problems that arise for eaters opting out of the general diet to adopt a personalized one in a country whose social representations of food revolve around the notion of food sharing.	1) The average increase of all research topics over time reflects a hypertrophy of communicational flows about food. Still, this general increase is not repeated in all subjects in isolation, which demonstrates that food/eating is a subject that responds to changes in the social context (dynamicity of communication flows). Regardless of the date, obesity, anorexia and "going on a diet" remained at the top of google searches.<br>2) In the last decades: ↑ the medicalization and individualization of food in rich countries. Background: [1] personalization of food "products" by the industry (food increasingly "consumable"); [2] ↑ Government Concerns (↑ NCDs). [3] New moralization of bodies (the duty to take care of appearance). [4] Banalization of diets. Such processes and background pose obstacles to commensality (not eating like others pulls you away from the table). "Food sharing practices have transcended a purely corporeal experience and have entered the digital domain (communities, blogs, photographs, diet sites...). Across technological and ideological changes food still remains, in France, an important instrument of sociability".	Formation of online communities in a context of construction of food identity from personalized diets.
Computational commensality: from theories to	2019	Radoslaw Niewiadomski,	Review Article	To discuss psychological and sociological studies on the social aspects of food and eating activities, showing	There are prototypes that facilitate food detection and recognition, that serve as artificial companions, virtual and augmented reality and to create haptic experiences. Also, there are technologies that enhance	Use of technological prototypes during meals

computational models for social food preparation and consumption in HCI		Eleonora Ceccadi, Gjis Huisman, Gualtiero Volpe e Maurizio Mancini		how they can be exploited to create CC; and (2) To present computational models, devices, and applications focusing on their social dimension, illustrating how they could be used in CC scenarios	flavor experiences.<br><br>The technology also appears in serious games and playful interactions around food and tele-dining.	
Cook it, eat it, skype it: mobile media use in re-staging intimate culinary practices among transnational families	2019	Sara Marino	Qualitative	Explore how immigrant women in London use culinary practices to re-establish connections through sharing recipes and cooking food remotely; in addition to creating and substantiating the concept of "transconnective habitus"	Re-establishing family rituals at a distance: (1) the participants used many technologies to support their communication around food, according to convenience and situation. (e.g.: Skype was associated with "needy" moments).<br>The interactions served for practical purposes — helping to make a recipe — and for intimacy — food as a pretext for interpersonal relationships. Skype was characterized as a medium and food as content to recreate the family relationship.<br>The relationship does not remain the same: there were reports of improvements on the family members' relationships, associated with sharing recipes, which served as a common link in evidence between family members.<br>Commensality and transnational social capital: (1) Skype is used to record important moments of socialization, as well as moments of commensality. (2) the use of food as a value and knowledge serves for Italian women to affirm their cultural identities (3) the return to Italian cuisine as a discourse that strengthens ties with the family. (4) Skype is also used for online mealtime socialization: at typical family Sunday lunches in Italy and at celebrations. On these	Sharing photos, exchanging recipes and making meals remotely from various digital tools, especially Skype

					occasions, the centrality of food and the use of technology arouse emotional closeness, which is a key element of moments of celebration. The use of such a practice in daily life is hampered by the work routine and time differences (the time zone problem is also observed).<br>Feeling alone, even if connected: the work routine might leave little time to call the family, the time zone appears as a challenge and the whole relationship depends on the parents' ability to use digital tools. Still, the positive results of the practice of digital commensality also bring nostalgia, feeling of loss of important occasions and "guilt" related to distance.	
Designing playful technology for young children's mealtime	2019	Ying-Yu Chen	Mixed methods	To examine current use and potential use of technologies as a form of entertainment among children and their families during meals	Participant observation: teachers wanted children to remain seated; children wanted to feel comfortable. Teachers encouraged the use of utensils; children wanted to eat independently. Teachers wanted to keep the table clean; children wanted to break food in an unconventional way, use their hands and mix things up: they wanted to eat creatively. Parents, on the other hand, wanted to encourage healthy eating habits, want to increase their children's food consumption, teach good table manners and talk.<br>Prototyping: the stamp plate, a prototype with a screen that allows the replacement of food by virtual pieces (bread as a brown square; cheese as an yellow triangle; and apple as a red half-circle) that could be freely rearranged in the form of drawings as soon as children finish their meals was listed as the prototype with the greatest potential, since it was able to contribute to the children's focus on food, offered tools for the exercise of creativity and regulated their behavior, according to the wishes of parents and teachers.<br>Empirical analysis: placed in a real family meal	Both traditional meals and meals with the Stamp Plate device were performed, all involving families with preschool-age children and their families.

					situation, the stamp plate contributed to a greater acceptance and interest in the foods offered (bread, cheese and apple), compared to the experience in a conventional meal. Also, when the children finished eating and realized that they needed more pieces to complete their drawings, they asked for more food. It was also observed that, when building their drawings, they imagined how other foods could be converted into useful and interesting pieces to complement their games, arousing spontaneous interest in other foods. In contrast, parents were able to guide their children's experiences, encouraging them to eat to gather pieces and controlling the pace of their chewing. Also, if children were in doubt about how to use the device, parents were able to promptly clarify them. The production of pieces, in addition, raised dialogues between parents and children about the amount of food ingested: since, at the end, for each triangle, there was a piece of cheese consumed and so on. This activity helped to ensure the children's attention to food and allowed reflections to be made. Finally, the use of the device was used to practice basic math and generated fun at the table.	
Digital Commensality : Eating and Drinking in the Company of Technology.	2019	Charles Spence, Maurizio Mancini e Gijs Huisman	Review Article	To provide an overview that serves to observe digital commensality	Problems Associated with the Growth of Living Alone: From the 1960s until now, the number of single-person households has grown 5x in the US. Worldwide, it went from 153 million (1996) to 277 million (2011). In a British survey, it was observed that 15% of those consulted did not have any meal with a family member in the previous 6 months, 30% also did not eat with their best friends in the same period and the same happened with 45% of the participants in relation to old friends. . As early as 2001, 3/4 British families had abandoned regular	Mukbang, skeating (meals shared via video call [synchronous or otherwise])

					meals together. This growth of solo eating exposes more and more people to problems such as cultural disconnection, while authors point out that many people do not consider solo meals as real meals and that the idea of an ideal meal still permeates the notion of having company to carry it out. there. Different eating companies generate different effects (negative or not) on different subjects: eating with the family, considering certain measures, is positive for social, health and weight parameters. At the same time, there is more evidence for this type of effect in children and adolescents. At the same time, eating alone is associated with stress, worse quality and higher food consumption and a decrease in the feeling of well-being. Also, people who live alone tend to waste more food than those who live with other people. Benefits and costs of eating with company: eating together is not always good, there are risks (such as the practice of excessive eating when in company). On the other hand, there are several benefits associated with commensality: food has been observed to sharpen our sensory apparatus more when shared: good tastes improve and bad tastes worsen. Furthermore, in one survey, 60% of participants reported preferring to eat while talking. In another research, it was seen that people trust strangers who share meals with them more. Still, there is potential financial benefit to sharing meals. Eating alone in public — losing the stigma: the practice is growing and gaining contours of social acceptability. During such meals, the use of electronic devices allows for distraction and interaction with geographically distant people.	
Does watching mukbangs help	2019	Wenzhuo Xu	Quantitative	To empirically examine the effects of mukbang on dieters	US Study: Satiety: Women in the mukbang group had 105.2% less satiety than the no-food video group.	People watching mukbang videos

you diet? The effect of the mukbang on the desire to eat				and to investigate the differences between food shows and mukbang. Also, offer elements for the debate about people who intentionally use "food cues" to control their appetites. Finally, test the effects of mukbang on the desire to eat, with a view to public health implications, if so	Those on diets ("dieters") had 212% more satiety compared to the rest. Also, dieters who watched the mukbang video had 15.9% less satiety than dieters who watched the video without food. Desire to eat: mukbang increased the desire eat dim sum as well as fruits or<br>vegetables and that dieters had less desire to eat. Factory Workers Study (USA): Satiety: Watching the donut mukbang video decreases satiety by 67% compared to the no-food video. Conversely, the ramen video increased the satiety of dieters by 104.3% and generated 47.9% more disgust than non-dieters. Dieters who used to watch mukbang had even more satiety (139.8%). Desire to eat: Ramen mukbang increased the desire to eat ramen by 428% compared to the no-food video. The donut increased by 228%. The donut video did not interfere with the urge to eat ramen or vice versa. The use of mukbang to replace consumption: seeing mukbang for dieting was associated with both greater satiety and greater disgust with food, which was considered a positive result in relation to the idea of a dietary strategy based on seeing mukbang, although it also has It was found that aversion and satiety did not diminish viewers' desire to specifically eat the foods shown in the video. Participants who successfully adhered to the mukbang diet had a greater desire to eat fruit and a lower BMI, associated by work with a greater level of self-control. China Study: None of the food videos caused disgust → participants used to mukbang videos. Likewise, there was no significant effect on diet control by exposure to video content. Still in the same direction, the frequency with which the participants saw mukbang did not significantly interfere with the results. Both food videos increased the urge to eat the	

					food shown on screen. There was no strong evidence about the potential for mukbang to cause satiety or satiety in participants.	
Eating and togetherness in “long distance families”	2019	Luna Fisker	Qualitative	To explore how technology can help families — whose members are physically far apart — feel close and formulate a prototype capable of fulfilling that role.	Commensality: what it is like to be apart and to be part (ethnography):<br>The experience of having meals mediated by video calls was pleasant and considered as close to a face-to-face meal as possible. However, due to the physical restrictions of the positioning of the phone, only a few people could see the author at a time (cell phone in the center of the table). The time dedicated to the meal was also posed as a paradoxical issue, while the time investment for making calls with the family was qualified as high, which could hinder the construction of social relationships in the place where the remote diner is located. fact.<br>Video calling involves a physical component: relatives wave to others, address others, change their cell phone position...<br>Finally, two areas needed to be defined for the meal: one in each house, making the space an important vector of messages and, therefore, a way to communicate.<br><br>Commensality and long-distance (semi-structured interview):<br>Emotional differences: the mother focused more on meals and food when she had them with her daughter. Using technologies while eating alone, the mother felt distracted and away from food.<br><br>Placebo sleeves:<br>synchronous communication:<br>3 of the participants, including the two design	Using a device during a meal to remind a loved one who is not there

					students, stressed the importance of timing meals. Furthermore, they highlighted the difficulty of precisely matching the beginning of meals (food needs to be ready at the same time) and also problems with routine and time zone differences.<br><br>Practical communication:<br>The same three participants suggested the use of color, vibrations and "squeezing" during communication with the placebo sleeves.<br><br>As a reminder:<br>all participants	
Eating together multimodally: Collaborative eating in mukbang, a Korean livestream of eating	2019	Hanwool Choe	Qualitative	To examine, from a conversational analysis, how the social dimension of eating is built together and in various ways (verbal and non-verbal) in mukbang communities. Or: how does the use of different languages favor collaborative eating in Mukbang communities?	The author identified 3 forms of multimodal interactions that contribute to collaborative eating: 1) eating through recruitment, e.g.: ChangHyun starts live (footing) and chat people "recruit" the BJ to eat different dishes, or when he trades tasks with the chat (chain of tasks). This category points to a collective construction of the community from collaborative tasks, direct commands, generally linked to the act of eating, mediated verbally and non-verbally.<br>2) Eating as a constructed action: animated embedding: the streamer establishes a (verbal) dialogue with a live participant and "stages" (action) their response (e.g.:, points food at the camera as if the other can reach it and then eats (incorporation), as if it were the other).<br>3) "Puppetization": a participant asks (verbally) for the streamer to eat (embedding) in a certain way and he responds (action. It is important to note that the streamer is not passive and can also create about the situation).	Live participants and a Mukbang streamer build interactional relationships (from speeches, written texts, performances, actions (such as donations), gestures, facial expressions, sound and visual effects) around eating together: all interactions, from somehow contribute to the sharing of food between those in front of the cameras and those who follow.

FObo: towards designing a robotic companion for solo dining	2019	Rohit Ashok khot, Harashitha Kurra, Eshita Sri Arzae Yan Wang	Report	To introduce the prototype of the FObo robot, a robot that behaves like a diner, and reorient the perception that robots don't always have to be infallible, they can also be messed up and fooled.	FoBo is still a work in progress and not all the functionalities (MIMICKING DINERS, BULGING OF THE BELLY, BURPING AND PURRING AND EATING) are fully implemented as yet. However, through FoBo, we aim to initiate a research focused critique and a discussion over the form and the functionality of a playful dining companion. With the gathered feedback, we aim to finish the development of FoBo, and then study its use in a variety of different solo dining contexts, that include home, restaurant and the workspace.	Robot meal sharing - virtual dining companion
I like watching other people eat: A cross-cultural analysis of the antecedents of attitudes towards Mukbang	2019	Beulah Pereira, Billy Sung e Sean Lee	Qualitative	To understand why the two proposed groups ("Asians" and "Caucasians") consume Mukbang	1) Attraction to the host positively influences participants' attitudes towards Mukbang. 2) Voyeurism, despite other studies, proved to be something that alienated Asian audiences from the Mukbang and was irrelevant to Caucasians. 3) The perception of novelty positively attracted the attention of the Caucasian public, while the opposite occurred for Asians. 4) The feeling of loneliness did not bring the Mukbang groups together, contradicting previous findings that pointed to the relief of this feeling sought by the Mukbang audience. 5) There was no perceived relationship between the participants' health perception and the willingness to watch Mukbang videos, contradicting the findings that put food porn and food shows as "escapes" for individuals who are dedicated to maintaining diets or " healthy lifestyles". 6) The feeling of collectivity was not understood as a motivating element among the research participants. 7) social norms contribute positively to the participants' relationship with Mukbang. Finally: in general, perceptions and attitudes around Mukbang positively impact the intention to attend Mukbang in both groups.	Diners watch VJs consuming large amounts of food, either via live streams or recorded videos shared on the internet.

Mukbang Influencers—Online eating becomes a new marketing strategy	2019	Mengyu Yang; Greta Babenskaite	Qualitative	To investigate brands' perspectives on marketing through mukbang, their practical concerns and factors that influence companies' decisions about using mukbang influencers as a marketing strategy	All companies responded positively when asked about the mukbang phenomenon. When asked if they have used or would be interested in using mukbang influencers, 3 of the companies said they did not consider using it, due to 3 reasons: fear of uncertainty; concerns about the effectiveness of mukbang marketing and concerns about the lack of ability to control the organization of the entire mukbang marketing process.<br>At the same time, one of the 5 remaining companies had already used mukbang as marketing and the others said they were considering it. The motivations for doing so lie in the desire to accumulate more information from the experimental use of mukbang marketing; use a new marketing channel and reduce the risk of the operation; enjoy the low price of mukbang marketing.<br>Brand concerns about the practice: lack of mukbang information, data and resources; fear of regulation and control; difficulty controlling influencers and mukbang marketing processes; relational difficulties with influencers and platforms. Yet, mukbang has not yet reached a considerable scale.<br>Finally, companies were asked about the future of mukbang influencers and mukbang marketing. Despite the limitations pointed out, companies think that mukbang influencers will grow in the coming years. Still, some of them think that companies' investment in mukbang marketing will increase, especially among small companies.	Selling products during food broadcasts (mukbang)
Older adults eating together in a virtual living room:	2019	Dannie Korsgaard, Thomas	Mixed methods	To facilitate remote social eating for lonely older adults	The virtual environment seen from the glasses (living room) was described as lovely, elegant, beautiful and impressive.	Remote commensality mediated by augmented reality glasses that projected a virtual room and avatars of

opportunities and limitations of eating in augmented virtuality		Bjorner, Jon Ram Bruun-Pedersen e Pernille Krog Sorensen			Participants prepared cake for themselves (as previously agreed). The conversations took place around food and the virtual environment<br><br>Participants discussed the limitations and potentials of the technology used. There was a certain frustration for not being able to see the other's food (it was only possible to see an avatar of the other participants)<br>.	the other participants
Pass the iPad: assessing the relationship between tech use during family meals and parental reports of closeness to their children	2019	Justin J. Nelson	Quantitative	Tp extend previous research in the field of ICT from the investigation of the relationship between frequency of family meals and feeling of closeness with children, and also investigate the relationship between two types of digital technologies used during family meals (tv and other) with the feeling of closeness with the children.	The use of these other technological devices, including cell phones, during the family meal is negatively associated with parental feelings of closeness to their children (odds ratio of 0.876, $p < .01$). Every one unit increase in the use of other digital devices at the table by anyone in the family decreases parental reports of being close to their children by just over 12%. Interestingly, this independent effect also held for a subsample of parents that reported eating together as a family every day ($N = 1,396$). With all else equal, digital technology use during the family meal (odds ratio of 0.868, $p < .05$) had nearly the exact same negative relationship on reports of closeness for this group. This indicates that this negative relationship works independent of family meal frequency and is not a product of decreased family meal interactions.<br><br>Also, the author estimated an interaction effect of gender and use of other electronic devices during the family meal on parental feelings of closeness. Here, the interaction term is statistically significant and positive (odds ratio of 1.231, $p < .05$), indicating a significant<br>difference between mothers and fathers with respect to the relationship between device use	Family meals using electronic devices and televisions

					during the family meal and reports of closeness to one's children. In other words, these relationships are gendered. Findings indicate that technology use during the family meal is not related to mothers' reports of closeness to their children but is for fathers.	
Social food pleasure: When sharing offline, online and for society promotes pleasurable and healthy food experiences and well-being	2019	Monica Mendini, Marta Pizzetti e Paula C. Peter	Review article	The purpose of this paper is to introduce and define social food pleasure as a new conceptual framework that can promote pleasurable and healthy food experiences.	The authors conceptualize social food pleasure as "the enjoyment derived from the acts of sharing food experiences offline, online, and for society at large, that positively contributes to consumers' overall pleasure and satisfaction with consumer's food consumption". Moreover, the authors identify three key contexts of applications of social food pleasure. Sharing offline relates to the social activities that can help achieve pleasure with food. Sharing online concerns new media tools which allow for the connection between consumers and food to enhance food pleasure. Sharing for society considers the current pleasure of consumers derived from having a positive social experience based on food consumption.	Traditional commensality/sharing food images on social media
Understanding Parents' Perspectives on Mealtime Technology	2019	Ying-Yu Chen, Ziyue Li, Daniela Rosner E Alexis Hiniker	Mixed methods	To understand how parents can respond to new ways of organizing (designing) the space for family meals	Recordings:<br>Parents wanted their children to eat diverse and nutritious foods. They also wanted them to eat more food. In addition, they strive to educate their behavior at the table and value the moment at the table as a space to talk about the family routine and encourage the children's speech, with a view to their days and to the increase of their speech and language skills. vocabulary<br><br>Survey:<br>Smart objects:<br>Parents were more resistant to smart objects, due to concerns about technology dependence and increased distraction at the table. However, physical issues	Hypothetical meal situations using screens/amazon echo/smart objects

					associated with the objects also raised criticism: adding electricity to utensils sounded dangerous, for example.<br><br>Screens:<br>Some of the parents reported already using screens during meals: educational and children's programs or children's YouTube channels, which caused a familiarity effect with the ideas of the authors who used screens.<br>Despite this, the use of screens was also associated with an increase in distractions and dependence on technology.<br><br>Amazon Echo:<br>Associated with awkwardness, intrusion, and repositioning (parents complained about voice design ideas taking on roles at the table that they understand are theirs)<br><br>The most accepted idea was the screen that teaches children about healthy eating.	
Videodining in older adults aging in place: a feasibility and acceptability study	2019	Laura k. Barre; Srah Coupal; Tara Young	Mixed methods	We tested the acceptability and feasibility of VideoDining in older adults receiving Meals on Wheels (MOW) and explored whether it changed meal intake.	Average meal length was 39 minutes and 40% ate more than usual, 30% ate the same, and 30% ate less. Reasons for eating less included being nervous and eating when not their usual mealtime. All participants reported they would VideoDine again and companion diners rated the overall experience a 9.2 out of 10. Older adults are able to VideoDine with a new acquaintance and have a positive experience.	Being in the company of another person during the meal through technology
Virtual Commensality —Mukbang	2019	Julia Aucoin	Qualitative	To analyze the formation of communities around "sensory-based" and "eating-	1) "Chapter One showed that mukbang as a cultural product in South Korea relied on a series of infrastructures developed over decades through state	Participatory culture of online<br>food broadcasts: where

and Food Television				centered" Youtube videos	policies including public initiatives surrounding the economics and social impact of internet and communications technologies. These infrastructures, which include public Wi-Fi, transit and social networks, enabled the digital platforms like AfreecaTV that mukbang would one day rely on as forms of mobile television. They were also conducive to social infrastructures that fostered relationships between mukbang broadcasters and networks of food delivery persons and fans, creating the early conditions necessary for community formation as people began to connect in social relationships through this medium". 2) feelings and affects that circulate within ASMR and mukbang work to hold together diverse online fan groups who are dispersed geographically and socially, into their own distinct communities. 3) "Mukbang communities are sustained through their highly mobile nature, fostering a community that is at once open to change while also operating under a set of shared community practices and policies of self-governance. These affective practices are evidenced through the comments, 'likes', collaborations and social interactions that mukbang videos encourage between viewers and creators, and among viewers and fans.	viewers post comments and "like" images, sounds and the broadcaster's narrative while eating. Hosts sometimes respond in turn to viewers' comments and likes.
Watching Women Eat: A Critique of Magical Eating and Mukbang Videos	2019	Samantha Gillespie Dr. Jenna N. Hanchey	Qualitative	From a feminist perspective, to closely investigate how audiences react to public displays of women eating. Still, interrogate the power of thinness in North American culture.	Not much has changed in how we perceive the power of thinness — a hegemonic standard that serves to keep both men and women, though historically women, oppressed. The author demonstrated the prevalence of eating disorders and using Mukbangs shows to literally curb hunger. Audiences turned to the thin-bodied stars to imagine the sensation of eating, and more importantly the sensation of eating without gaining weight. This was done repetitively, and	Mukbang (Political-cultural community interactions around female mukbang stars)

					encouraged by viewers as a successful way to avoid cravings. Intertwined with thinness culture is the construct of gendered eating norms. These rules also have not progressed much throughout history. How the concept of magical eating contributes to these conversations is by looking closely at the functionality of people's fantasies of eating and embodiment, and the contradictions in their fantasies. In this work The author established that there is strong attraction from audiences to watching women eat--and particularly, watching the binge eat. In part this is a rebellious act. However, when scrutinized, the research shows, the draw to these texts is really about people's desires to escape their own internal contradictions over standards of eating and embodiment. However, magical eating is but a fantasy, not a way of traversing it.<br>Cap 3: the author was able to determine themes that focused on common points of struggles with food and cultural eating norms presented in Western societies. I argue that though Mukbang shows rebel against gendered eating norms, they also work to reinscribe belief systems of thinness culture because of how audiences react to public displays of women eating. In this chapter, it was demonstrated moments of the audiences supporting and encouraging the rebellious act of women eating in public spaces. However, also in this chapter I also point to ways the audience reinscribes thinness culture. I do this by observing language which both emboldens and restrains eating behaviors. Through these observations, it was possible to form themes focused on audience reactions to eating aesthetics, fantasies of indulgence, and desiring similar experiences with food.<br>Cáp 4: the author focused her work on how audiences	

					are responding to Mukbang stars with ideal and nonconforming body types. This is useful in understanding the audiences' fantasy of binge eating in relation to the presenting body featured in the fantasy. This work leads to a larger discussion of how thinness functions in U.S. culture and the Othering of fat bodies. The audience's discourse encouraging Mukbang stars to engage in rebellious acts seen in Chapter 3 departs from the narrative in this chapter. The patterns that was determined functioned to strongly reinscribe ideologies which privilege thinness and actually erasing fat identity (LeBesco & Braziel, 2001) all together.<br>Cap 5 (comer mágico): she connected her work from chapters 3 and 4 to explain the functionality of magical eating. I illustrate how the immediacy of this logic emerges from a need to undermine the historic constraints placed on women for decades dictating unattainable and oppressive eating norms and expectations of their bodies. These texts create a false sense of relief for audiences from these norms. However, in using this form of magical thinking, there also are ways that harmful ideologies continue to be reinscribed, defer any real introspective on one's own damage from the repercussions of thinness culture, and abilities to transform eating and thinness culture all together.	
"The Toad Wants to Eat the Swan?" A Study of Rural Female Chibo through Short Video Format on E-	2020	Lilin He	Review Article	Investigate how the phenomenon of "Rural female Chibo" presents itself and the deep reasons that motivate rural women to practice it.	Most of Chibo's anchors are women, corroborating the interest of the majority of the male audience. The most popular platforms among Chibo's consumers are TikTok and Kuaishou.<br>Chinese e-commerce services such as Taobao and T-mall have also added short video posting functions to their platforms, riding the wave of popularity of Chibo	Relation of the Chinese community with the transmission of Chibo.

commerce Platforms in China.					and also showing details of services and products to consumers. In summary, Chibo broadcasters serve as intermediaries between sellers and buyers or ambassadors of food industry brands in China.<br><br>The development of the scenario of female influencers in rural areas enabled the rise of figures who were out of harmony with the current standard of beauty and who had less schooling, besides the criticism and aroused discontent in part of Chinese society.<br>Established in the Chinese digital landscape, these influencers enjoy a high level of connections and opportunities.<br>The Chibo's video makers commonly use "sexy and smooth" voices as a way of captivating a larger audience and receiving gifts, in a movement of implicit pornography as a fast-growing strategy.<br>In Kuaishou, there is a particular type of chibo produced by rural women to rural chinese people. Those women don't identify themselves with the Chinese patterns of beauty and urban "excessive use of makeup". In some cases, city women invest in a rural aesthetic to interact with the rural public.	
"Always on the table": revealing smartphone usages in everyday eating out situations	2020	Philip Weber, Philip Engelbutzeder and Thomas Ludwig	Mixed methods	Explore the practices that exist around the use of mobile technologies in the setting of meals	Using the cell phone before eating: the interaction with reviews of places on the internet.<br>We observed 30 cases in which the smartphone was used to take pictures of food (food porn) and most of them took place before starting the meal. This practice was sometimes seen as a powerful way of communicating with the world and sometimes as something fun, but unimportant.<br>Still, part of the participants (23) rejected or were indifferent to the practice.<br>Another relevant behavior pointed out that 10	Smartphone use before, during and after meals (eating out and eating alone), Food porn, traditional dining and interactions around food on the internet (comments on food websites).

					respondents prefer to send their photos to family or close friends by message, instead of posting them on Instagram<br>Using the cell phone during the meal: most of the time, served to encourage conversation between diners (show or share photos and memes; check news and facts related to the conversation and, to a lesser extent, use the calendar to plan your agenda) .<br>On the other hand, the smartphone was used by many participants to check notifications.<br>Among solo eaters, the smartphone is widely used for entertainment or as a means of communication with other people.<br>Despite the findings, 19 of the 29 participants judged the use of smartphones during meals to be negative. Concurrently, in the observational study, 41% of participants did not use smartphones during their meals.<br>Use of smartphones after meals: use of instant messages to talk about the experience to friends.<br>Among those who do not usually use their smartphone during meals, a feeling of need to update themselves was observed (see "missed" notifications and new events)<br>Smartphone use was also observed to check and analyze food consumption throughout the day.	
A Development of Color Lighting Device to Indicate Subjective Palatability	2020	Akira Kashihara, and Tomoyuki Yamaguchi	Quantitative	To develop a subjective palatability display device to facilitate emotional communication with family/friends during meals	A diner could indicate the subjective palatability to an observer using the proposed device with the same accuracy and speed of vocal pression. However, we could not get the result under our hypothesis. The reason why vocal expression is the same accuracy and speed of the proposed device is that voice includes tone and intonation. We assumed that voice could only express the word before this research, however, the	Lights represented by a device that emits the subjectivity palatability of those who ingest the meal.

					effect of intonation and tone can indicate the detail of the subjective palatability. The proposed device also can indicate subjective palatability. The color corresponding to the subjective palatability is clear so an observer can guess what taste a diner perceives.	
A study on potential health issues behind the popularity of “mukbang” in China	2020	Shan Wang	Qualitative	To explore the relationship between mukbang videos and viewers’ perception of food and health	Viewers' speculated on the quantity of food consumed by mukbangers when they are not in front of the camera. Also, they showed envy or the desire to own their "slim" bodies "that never become overweight" even with the mukbang practice. The body comments were genre-oriented (Chinese culture of thinness: control over shapes, language and behavior; and rejection of all kinds of transgression, like mukbang).<br>The audience expressed how the videos made them hungry and, at the same time, expressed satisfaction as seeing the mukbangers eat.<br>The audience felt close with the broadcasters and expressed their emotional connection with them. At the same time, they expressed their concerns about their eating habits, classified as harmful to health.<br>The audience also said that the mukbang culture could be influencing their eating habits.	Mukbang - a person eating large amounts of food while streaming.
Changing Roles and Contexts: Symbolic Interactionism in the Sharing of Food and Eating Practices between Remote,	2020	Aswati Panicker, Kavya Basu e Chia-Fang Chung	Qualitative	To explore the social dynamics around food in families that relate to each other remotely, focusing on changes in roles and contexts associated with distance	Develop awareness of the routine of the other: talking about food was seen as a way to be aware of the routine, health and well-being of a distant family member. In addition, a movement of role changes was observed between the participants and their families, as the spatial division of the family sometimes redefined roles of responsibility for food among its members. Food as a way of remembering past experiences and maintaining connection in new contexts: participants reported using food as a way to access past experiences and memories, in a way of	Sharing photos, recipes and holding video conferences

Intergenerational Family Members					showing affection and creating new experiences, such as sharing experiences. photos, recipes and making meals by video call.<br>Healthy eating and caring for the health of the other: participants reported feeling motivated to talk about the food of the other, in order to demonstrate and practice care, especially if a family member had any concerns or worsening in relation to the other's health. your health status. At the same time, conversations about healthy eating were characterized as a subject conducive to tensions, since different family members have different perspectives and levels of interest on the topic.<br>Accountability for healthy eating due to role changes: on certain occasions, some participants started to share healthy eating practices after they perceived themselves in new roles within the family dynamics, such as after the birth of grandchildren and with the aging of parents. However, such situations were also a typical start of tension between family members, which could be mitigated during the process of transition of roles within the family dynamics. Associated food preparation, gender and roles in family dynamics: when together, family members usually depended on the same figure to take care of all the food in the house. Separated, family members began to turn to this same figure whenever they wanted to learn recipes or help with food. The authors point out that this figure is mostly female.	
Development and Validation of the Mukbang Addiction	2020	Kagan Kircaburun, Vasileios	Quantitative	To investigate negative effects of Mukbang from the development of a Mukbang dependence scale.	The scale works as a valid psychometric tool to measure problems related to the act of watching mukbang. The daily frequency of participation in mukbang communities is directly related to a problematic development of the practice. Unlike the	Mukbang - a person eating large amounts of food while streaming.

Scale		Stavropoulos, Andrew Harris, Filipa Calado, Emrah Emirtekin e Mark D. Griffiths			Facebook addiction scale study, there was little occurrence of cases in which everyday activities such as studying and working were negatively impacted because individuals replaced them with hours of Mukbang.	
Eating alone, or commensality redefined? Solo dining and the aestheticization of eating (out)	2020	Sami Koponen e Pekka Mustonen	Qualitative	Demonstrate how eating alone in public correlates with new forms of togetherness	Dining alone versus eating together: commensality redefined? Solo dining was approached as a culture of togetherness, based on the understanding that commensality can take place between the diner and restaurant workers or other diners (present or not). The commensal act would surround the sharing of aesthetic values in relation to food and drink. Such sharing can be observed in the choice of restaurants (in addition to good service, they must dialogue with established values of gourmet food culture, while those that do not usually generate a solitary experience among solo diners. Nevertheless, solo diners can practice commensality with their peers through the use of digital technologies, before, during or after foodie experiences.	Interactions with employees or other solo diners using digital technologies
Eating as a transgression: multisensorial performativity in the carnal videos of	2020	Yeran Kim	Qualitative	Identify the ambivalence, excessive content and complexity inherent to the operation of mukbang on social connection platforms, in addition to positioning	Definition of carnal videos, based on Deleuze and Guattari's concept of "fleshism": a pious and sensual expression of excess, dynamics and rupture of the flesh as a block of sensations (art) in the contemporary social media environment. Furthermore, this expression was captured by the power algorithms of	Construction of a network of affections between machines, language, objects, social and economic structures and human beings around the

mukbang (eating shows)				mukbang, based on its social impact, and capitalism, based on its political-economic control forces over mukbang practices. The ultimate objective is to attest to the conflicting and protesting nature of the reality of what the author defines as carnal videos.	digital platforms, operating in a structure that imposes subversive roles on anonymous people, according to the characterization of the control society.<br><br>The multisensory virtualization of the act of eating:<br>Mukbang is a cultural way of post-capitalist societies to deal with the act of eating from a perspective of multimodal production, circulation and consumption (sounds, tastes, images and forms of language).<br>The mukbang aesthetic is characterized as instinctive, wasteful, lonely and challenging and its BJs (broadcasters) operate it during their performances. Nevertheless, the mukbang makes a cyclical movement of transmutation from what is desirable to be rejected and from pleasure to pain.<br>From a cultural and social perspective, he subverts political, cultural and social codes imposed on bodily practices.<br><br>The affective vortex of disgust at mukbang<br>A series of discursive diagnoses have pointed to explain psychological aspects of mukbang consumers point to the practice as an alternative way to obtain satisfaction in the face of social and existential problems associated with reality, especially among young people (as is the case of young women who attend mukbang as a form of to oppose the pressure for a thin body).<br>This characterization explains the mukbang's potential to access lonely and desolate young people, playing a role in the midst of efforts to satisfy emotional hunger and feelings of despair, loneliness, isolation and loss. In short, there is the construction of a network of affections between machines, language, objects, social and economic structures and human beings.	mukbang

					In this way, the initial impression of greed and disgust attributed to the practice and BJs is replaced by confidence, pride, and pleasure. Such a configuration makes shame and insults function as a resource with the potential to be turned into fame and popularity, characterizing BJs as ambiguous subjects: between pleasure and disgust.<br><br>Capture and control around mukbang<br>The mukbang is crossed by the economic and political conditions of capitalist society.<br>BJs put themselves in work situations for free and, in different ways, contribute to the capture of data, which serve to move the businesses of the companies that control digital platforms.<br>Since mukbang involves bodies, affections, senses, symbols, values, data and networks, it is possible to say that it involves life itself. Going a step further, in the context of the relationship between mukbang and capitalism, it is possible to classify it as a form of biowork, in which life is put in function of work, in a process of exploitation and monetization.	
Eating with an artificial commensal companion	2020	Patrick Gallagher, Radoslaw Niewiadomski e Merjin Bruijnes, Gijs Huisman	Mixed methods	To investigate whether participants prefer the interactional and social presence of an Artificial Commensal Companion to eating alone and whether it can influence food choices solely through their non-verbal behavior	Quantitative results: in the base scenario, 59 chocolates were consumed from the left bowl and 58 chocolates from the right bowl. In the active scenario, 61 chocolates were consumed from the left bowl and 53 chocolates from the right bowl.<br>Qualitative results: all participants reported liking the robot. One of the participants also mentioned enjoying the presence of the robot while he ate the chocolates.<br>All participants said they enjoyed the taste of chocolates more during the active scenario.<br>Asked about the possibility of using the robot during	People ate in front of a robot that interacted verbally and non-verbally.

		e Maurizio Mancini			meals, 4 of the participants said they would only do it if there were more people present. Participants who considered having the meal alone with the robot highlighted its potential in terms of entertainment, while the predominant suggestion regarding the robot's functioning called for the addition of the ability to talk.<br>3 participants believed that the most benefited from the use of the robot would be people who live alone.<br>Still, only one participant mentioned the power of influence over the choice of chocolate bowl, while the others thought that its function was merely to entertain.<br>Finally, 4 of the 6 participants mentioned that they liked the chocolates in the target bowl more, presented during the active scenario.	
Guess who's coming to dinner? surveying digital commensality during covid-19 outbreak	2020	Eleonora Ceccaldi, Gualtiero Volpe, Gijs Huisman, Maurizio Mancini	Quantitative	To investigate the use of technology for commensality during the covid-19 pandemic lockdown	People choose to eat online motivated by diverse feelings and intentions: not just to get rid of boredom and loneliness, but to feel closer to their friends they can't find.<br>Those who decide not to eat online don't feel that the use of technology does not allow them to be in company with family and friends.	Eating using skype
La hospitalidad en la escena gastronómica: sitios de internet de comida	2020	Maria Claudia Gavioli e Sênia Regina Bastos	Qualitative	To characterize the gastronomic scene and its actors and to differentiate the domestic environment from a commercial hospitality environment, in addition to demonstrating how	The article was able to highlight the ability of food airbnbs, through hospitality, to generate interest from amateur chefs, tourists looking for local food and immersive experiences, food enthusiasts, investors and people open to new experiences; and that, promoting the meeting of these actors, ultimately, is to feed an exchange of social signs, subjectivities and	Chefs and guests gathered through websites shared meals and gastronomic experiences

compartida [Hospitality in the gastronomic scene: Shared Food Websites].				hospitality is represented in new commensal businesses promoted by shared food internet sites.	identities.	
Mukbang-and cookbang-watching status and dietary life of university students who are not food and nutrition majors	2020	Sowon Yun, Hynjoo Kang, Hongmie Lee	Quantitative	To identify patterns of observation of Mukbang or Cookbang and their possible association with the eating life of university students who are not majoring in food and nutrition.	Korean college students who tend to attend Mukbang more often may have less nutritious diets, and are more likely to prefer to eat out or buy ready-to-eat foods. For those who watch cookbang, it was more related to wanting to cook their own food.	Mukbang - a person eating large amounts of food while streaming.
Mukbang and Disordered Eating: A Netnographic Analysis of Online Eating Broadcasts	2020	Mattias Strand and Sanna Aila Gustafsson	Quantitative	To explore how Mukbang viewers relate the practice to their eating disorders	There is an ambivalence in the relationship with Mukbang illustrated in the comments: mukbang, in general, presents positive and negative implications at the same time, and can arouse different affections and behaviors simultaneously. Viewers develop "parasocial" relationships with mukbangers, in a way that accompanying them is often understood as a way to feel less guilty and lonely. However, how this approach to sociability reflects on the way viewers perceive and relate to their bodies remains ambiguous. A relationship between excessive consumption and the desire for a certain social status based on consumption and the availability of pleasure can be linked to Mukbang (as well as spectacle, hyperreality, virtuality and the new ecology of media). Mukbang as food porn: "ingestion without incorporation".	A presenter practices mukbang in YouTube videos, while part of his audience varies their eating behavior based on what is watched: sometimes eating more, sometimes eating less, sometimes feeling a certain feeling in relation to what is eaten by the presenter or by himself, sometimes feeling another... As well as mediations linked to food porn.
Problematic	2020	Kagan	Quantitative	To examine the relationship	The analysis indicated that problematic mukbang	Consumption of mukbang

Mukbang Watching and Its Relationship to Disordered Eating and Internet Addiction: A Pilot Study Among Emerging Adult Mukbang Watchers		Kircaburun, Cemil Yurdagul, Daria Kuss, Emrah Emirtekin e Mark D. Griffiths		between problematic mukbang consumption with eating disorders and internet addiction	watching was positively correlated with disordered eating ($r = 0.24$, $p < 0.01$) and internet addiction ($r = 0.26$, $p < 0.01$). However, the correlations were relatively low.<br>Problematic mukbang watching was positively associated with both disordered eating ($\beta = 0.43$, $p < 0.01$; 95% CI [0.10, 0.47]) and internet addiction ($\beta = 0.29$, $p < 0.01$; 95% CI [0.13, 0.70]). Problematic mukbang watching explained 18% of the variance in disordered eating and 9% in internet addiction	videos on video platforms
Quarantined Sobremesa	2020	Gabriela Aquije Zegarra	Qualitativo	To Create ways to adapt the dessert for digital platforms	The event settings had to be adjusted to accommodate the geographic (temporal) differences of the participants.<br>Held during the Covid-19 quarantine, the meals served to take diners out of the routine and provided the opportunity to hear about the isolation experience from the perspective of people from other countries.<br>Repeating the face-to-face realization of desserts, in the quarantined dessert, the hosts only had control over the configuration of the tables, while the subjects and behaviors depended on a joint construction between all the diners.<br>Nevertheless, the digitally created space particularly stimulated discussions about food systems and their relationship with the pandemic, in the conformation of a kind of global table.<br>Ultimately, food served to connect not just people to people, but people to cultures and food networks.	Use of videoconferencing tools to hold meals between geographically distant diners
Reflection:	2020	Irene	Qualitative	To explore how participants	By qualitatively analyzing the reviews left on the most	Remote hosts offered dining

Airbnb's food-related "online experiences": a recipe for connection and escape		Cenni, Camilla Vásquez		in Airbnb's online experiences perceive it and conceptualize the practice.	accessed pages of Online Experiences, the authors were able to state that one in three people spontaneously associated the experience with the feeling of commensality and that one in five said they intended to repeat it. Furthermore, the video provoked a feeling of closeness between the participants and the dynamic was effective in promoting socialization.	experiences to remote guests via the Airbnb platform
Room for one more? Introducing Artificial Commensal Companions	2020	Maurizio Mancini, Gjis Huisman ,Radoslaw Niewiadomski, Merijn Bruijnes	Qualitative	1) to build an artificial commensal companion (ACC) that is able to create a meaningful interaction with humans while eating and 2) to measure and replicate with ACCs at least some of the benefits of human-human commensality	The text points out the functionalities planned for the robot (human diner tracking; signal planner, so that the robot emits non-verbal signals during the meal; eyesight model; emotional feedback, a function that allows the robot to react positively when the human commensal takes the food it prefers; and movement functions)	Commensality between robot and human
The presence of smartphones at dinnertime: a parental perspective	2020	Hasan Latif, Aysen Simsek Kandemir, Seher Uçkun, Engin Karaman, Asiye Yuksel e Omer Alperen Onay	Quantitative	To examine children's smartphone use and children's e-communication behaviors from their smartphones while having dinner with their families	Smartphone use negatively affects the importance families place on meals. At the same time, the priority given to smartphones positively affected meals	Family meals involving smartphone use
The popularity of eating	2020	EunKyo Kang,	Quantitative	To analyze the content of mukbang videos on Youtube	Most videos do not involve food preparation and show broadcasters eating excessive amounts of food. Still, a	Mukbang - a person eating large amounts of food while

broadcast: content analysis of "mukbang" youtube videos, media coverage, and the health impact of "mukbang" on public		Jihye Lee, Kyae Hyung Kim e Young Ho Yun		and of news about mukbang published in newspapers, in order to explore the relationship between mukbang consumption and healthy habits	minority show consumption of irritating, spicy or dangerous/unhygienic food.<br><br>About the type of food consumed: most videos do not involve consumption of junk food, alcohol and instant food.<br><br>People who always watch mukbang reported that the practice has a strong influence on their eating habits.<br><br>Most newspaper articles explored the "fun" side of mukbang	streaming.
The Psychology of Mukbang Watching: A Scoping Review of the Academic and Non-academic Literature	2020	Kagan Kircaburun, Andrew Harris, Filipa Calado, Mark D. Griffiths	Scope Review Article	To examine the psychological characteristics of mukbang viewers and consequences of mukbang watching.	Social use: (1) one of the most noted aspects of the mukbang phenomenon was its role in social facilitation; (2) mukbang videos had the potential to counteract loneliness and isolation by connecting and sharing a similar interest with a virtual community; (3) mukbang can be used to psychologically facilitate commensality in order to cope with eating alone; (4) mukbang can foster feelings of affective connection with other individuals... "Sexual Use": fetishization of women eating. (1) slim and attractive female mukbangers were usually surrounded by overweight male fans and viewers; (2) host attractiveness was positively related to attitudes towards mukbang. Entertainment use: Individuals who watch mukbang also seek entertainment. Escapist Use: some studies have theorized that viewers use mukbang watching as an escape from reality. Eating Use: "magical eating fantasy"; (1) individuals watched mukbang to have the experience of eating vicariously through mukbangers because they were on diets; (2) proximity to food porn, webcam porn (for some viewers, the mukbangers were	Mukbang - a person eating large amounts of food while streaming.

					"prostitutes that eat"). Consequences: (1) mukbang could promote problematic eating and food practices among both mukbangers and viewers for those who were already experiencing different eating problems; (2) mukbang videos also affect viewers' perception of food<br>consumption and thinness.	
Towards experiencing eating as play	2020	Florian "Floyd" Mueller, Yan Wang, Zhunying Li, Tuomas Kari, Peter Arnold, Yash Dhanpal Mehta, Jonathan Marquez, Rohit Ashot Khot	Qualitative	To structure the understanding of how to create interactive technological systems that allow the act of eating while having fun	1) Make eating a challenge:<br>Arm-a-dine: limits the ability to eat; adds effort to select the food on the plate<br>YBEtS: removes sight of food/limits ability to eat<br>Edipulse: delays the time needed to eat<br>Tasty Beats: delay time to eat<br><br>2) Breaking cultural norms around eating:<br>Arm-a-dine: Breaks the rules of table etiquette<br>YBEtS: facilitates the act of feeding each other and increases the amount of spilled/dropped food<br>TastyBeats: Increased spilled/dropped food<br>LOLLio: facilitates the consumption of unwanted foods<br>Chewing Jockey: Breaks the Rules of Table Etiquette	Eating while using prototypes
Why do people watch others eat food? An Empirical Study on the Motivations and Practices of Mukbang	2020	Laurensia Anjani Terrance Mok Tony Tang Lora Oehlber	Qualitative	Find out about practices and motivations around watching mukbang.	Most of the sample reported that they watch the mukbang videos alone, either out of shame or because they consider something very personal (almost like porn). It is also used as a form of companionship during meals (often the videos are free, so it gives the aspect of dialogue); mukbang spectators report that it is a pleasurable activity that relaxes them. Over the motivations is placed the sense of spectacle,	Mukbang

Viewers		g Wooi Boon Goh			connectivity and considerable pleasure in watching the videos.	
Sharing Virtual Meals Among the Elderly: An ethnographic and quantitative study of the role of smartphones in distanced social eating in rural Japan	2020	Rise Sasaki, Laura Haapio-Kirk, Yumi Kimura	Mixed Methods	To propose a nutritional intervention through the use of smartphones in elderly people	The intervention in the chat group generally demonstrated a positive impact on the participants' mental health and eating habits. The creation of the group encouraged the sharing of photos of their meals and crops, as well as recipe tips, among others.	sharing photos and food emojis
A human voice, but not human visual image makes people perceive food to taste better and to eat more: "social" facilitation of eating in a digital media	2021	Nobuyuki Kawai, Zhuogen Guo e Ryuzaburo Nakata	Quantitative	(1) Assess whether the presence of a human voice without a video (as in radio) facilitates solo eating as much or more than a video of a person performing an activity unrelated to eating without audio. (2) Assess whether a video without audio of a group of four people facilitates solo eating more than a video of a person without audio. (3) Whether a non-human sound stimulus also induces food consumption and food taste perception.	Experiment 1: Participants rated the two items with human voices higher.<br>Participants ate more popcorn in human-voice situations.<br>Experiment 2: Participants rated the two items with human voices higher.<br>Participants ate more popcorn in human-voice situations.	Eating alone while watching a video/listening to an audio.

Aesthetics of Food Porn	2021	Uku Toomin g	Opinion article	Understand Food Porn's broad sharing appeal and clarify whether it is capable of creating creative gustatory imagery	The pleasures that one can take in Food Porn (FP) are diverse and do not just consist in blind arousal. In fact, what FP is in a particularly good position to afford are pleasurable imaginings that construct a gustatory object in response to what is seen in FP images. FP makes thereby possible cross-modal gustatory experiences which it might not be possible to have with the food that was the causal source of the image. This makes it more understandable why so many people are attracted to those images: they afford something like a (virtual) gustatory pleasure, not merely an enjoyment of the visual. What is more, FP images can also be evaluated in terms of their artistic merit, depending on the inclusion of secondary attractors and the sophistication of the gustatory imaginings that an image induces.	Sharing food porn images on social media.
Cloud-Based Commensality : Enjoy the Company of Co-diners Without Social Facilitation of Eating	2021	Chujun Wang, Yubin Peng, Linbo Qiu and Xiaoang Wan	Quantitative	To compare the consequences of Mukbang, cloud-based, and in-person commensality	Unhealthy foods were considered as being more familiar, pleasant, palatable, and energy dense than healthy food, but healthy food was considered more healthy. Healthy food scores of familiarity, pleasantness, and palatability were higher than the middle point of the used scale, which suggests that they also had those attributes.<br><br>In-person commensality elicited the highest probability of choosing unhealthy foods. Mukbang-based commensality elicited the highest probability of choosing healthy foods.	Mobile device
Contributions des Médias Sociaux aux	2021	Maxime David	Qualitative	Given the heterogeneity of discourses on what should be healthy eating, to explore	Respondents familiar with social media and interested in healthy eating most have developed specific power supplies. For example, many are	Social media

Représentations et aux Pratiques d'une Alimentation Saine chez les Jeunes [Social Media Contributions to Representations and Healthy Eating Practices in Young People]				how do young adults build and transmit their own representations and practices food, through social media	flexitarians, vegetarians or vegans. Also, a desire for self-control is observed in the speeches. Those with the most athletic profiles tend to follow a supplementation as part of their practice, with a view to improving their performance and recovery.<br><br>Social networks have an important informational role. If respondents have questions in relation to food, it is first through the search engines that they will look for some answers. However, the groups, communities, influencers to which they are subscribers, offer them daily information on the subject, contributing to improve your knowledge. But sorting information can sometimes be difficult. During hesitation about information, young women often rely on their own knowledge, their past experiences, their intuition, or even the confidence they grant to the source in question. The trust placed in an influencer is therefore essential to receive information.<br><br>Different factors that influence eating behaviors are highlighted. All first, the impact of influencers, who can be sports coaches, nutritionists, "fitgirls", or even journalists. The discourse of brands in the media it can also encourage people to adopt certain eating behaviors. Also, videos about the conditions of animal exploitation, can create a rupture in the way of thinking about their	

					food consumption. Finally, successful documentaries, some available at Netflix, others on Youtube, broadcast specific representations, often in connection with the promotion of vegetarianism. Many respondents said they liked this content, reinforcing their representations of food.	
Digital food culture, power and everyday life	2021	Zeena Feldman and Michael K. Goodman	Essay/Editorial	To present a short discussion of the social dimension of food and address a number of critical points about digital food culture post-COVID to suggest ways for further critical research	The pandemic changed how and where to cook, eat and hospitality are done. The dinner parties went virtual and people increased the use of video conferencing apps to eat together. Social media introduced people to new ingredients and recipes during the pandemic.	Social media
Investigating meal-concurrent media use: Social and dispositional predictors, intercultural differences, and the novel media phenomenon of “mukbang” eating broadcasts	2021	Jan-Philipp Stein and Jiyeon Yeo	Quantitative	To set out to gain a basic understanding of how different life circumstances and personality traits might predict people's inclination to use media during their food intake and investigate why people enjoy watching others eat (on a screen) during their own meals.	The data showed that South Korean participants expressed a more frequent use of nearly all types of media while eating, in particular concerning YouTube videos. In contrast to this, German participants indicated a slightly stronger reference for streaming services during their food intake, although this difference was not statistically significant.<br><br>42.2% of the Korean sample claimed to use social media every single time they ate food (indicated by the maximum value on the respective scale), followed by 41.5% watching YouTube during every meal. In contrast, much fewer German participants reported to use meal-concurrent media	Streaming platforms

					that excessively — even in the most popular category, only 6.7% of the sample claimed to always use<br>streaming services during meals.<br><br>German participants' meal-concurrent media use could only be connected to their age, whereas dispositional, social, and attitudinal variables did not offer a noteworthy explanatory contribution. A different pattern emerged in the South Korean sample: In this cultural group,<br>age did not influence participants' tendency to use media while eating;<br>instead, the self-reported inclination to show this behavior was linked<br>to a more extroverted personality.	
Comer com um robô para recuperar a confiança. Refeições, role-playing e comunicação no Japão [Eating with a robot to regain confidence. Dining, role-playing and communication in Japan]	2021	Agnès Giard	Opinion Article	To reflect on why people share meals with synthetic creatures or robots	In Japan, there is a rise of loneliness and of eating with robots and characters, which is stimulated by marketing, corporations, social media and engineers and design researches. This scenario supports a fake notion of decreasing the social problem of individualization of lives.	Robots and synthetic creatures
Share my meal: A Social	2021	Betzabe	Qualitative	The purpose of this thesis is	For all the participants, convenience, practicality, and	App

Catalyst for Interactions Around Food		Salinas Tovar		to investigate food practices national students in Stockholm, Sweden to provide an answer to the question: "How can we foster a<br>sense of connectedness among international students through everyday technologies?"	simplicity were common variables when it comes to food practices, specifically at the time of cooking. Food choices were not only influenced by the time taken to prepare the food, but also by what comes later, such as washing the dishes. convenience plays a key<br>role in food practices it may also cause participants to perform food practices only to comply with the basic human needs and detach from an experiential purpose. Planning was present in most of the routines of the participants as some prepared their food in advance and stored them for some days.<br><br>When the participants cooked food that they associated with<br>their home country, feelings of uniqueness, exclusiveness or<br>feeling special were evoked due to the ability to cook something from home, even with the constraints of being distant and finding the right ingredients. Sometimes just a single ingredient, rather than a complete meal brings memories or feelings of joy. Eating can also be<br>related to certain events not necessarily related with family<br>but rather with friends.<br><br>Food promotes interaction with other people by helping to<br>get to know each other's culture.	

					Prototype: was created an app that enhances creativity, gifting, trend-seeking behaviors and relaxation around healthy food.	
Understanding changes in behavior during the COVID-19 pandemic: Opportunities to design around new eating experiences	2021	Mario O. Parra, Luis A. Castro, Jesus Favela	Qualitative	To understand how lockdowns or self-confinement are shifting the eating experience for young professionals in Mexico.	Design ideas<br><br>In relation to negative feelings impacting eating patterns, our informants reported that during the pandemic they felt low, apathetic, stressed, and experiencing other negative emotions or states that provoked episodes of binge eating. Therefore, they envision applications capable of monitoring not only eating habits, but also users' emotional state, and offer recommendations.<br><br>The pandemic also caused our informants to cook and eat more often at home, since people were mostly at home and restaurants closed for eating in. If this becomes a habit, this could be beneficial since it favors healthy eating. Online interactions with friends could enhance cooking and make it more amusing for people.<br>Additionally, one of our informants reported about practices related to mindful eating. This practice predicts healthy behavior for people who use it daily.<br><br>The designs of the new technologies should focus not only on replacing face to face with virtual interactions, but also should consider what aspect of the eating experience interactive technologies can	Apps, social media and videoconferencig tools

					create, shift, or augment (e.g., food, taste, music, mingling).<br>In this matter, they envision ways for technology to design new environments for interaction while eating. During the pandemic, new applications for this kind of interaction started rising. For instance, a new platform called Gather Town (gather.town) allows users to create virtual spaces in which people can interact with virtual objects moving their avatar across the space. It also allows users to interact with other people simulating video calls (with cameras and microphones) when you are close to other avatars in the virtual space. These types of virtual interactions generate new opportunities for socializing while eating, for example, a virtual restaurant could be designed where people simulate their social activities before the pandemic.	
Asynchronous Co-Dining: Enhancing the Intimacy in Remote Co-Dining Experience Through Audio Recordings	2021	Huizhong Ye, Zengrong Guo e Rong-Hao Liang	Mixed methods	To create an "asynchronous co-dining" experience using recorded sounds	Increasing intimacy: most participants made sounds during meals, sometimes talking directly to the recipient of the recorded audio (active expressions), sometimes talking to themselves, with other people in the house or with pets (passive expressions), even though they could not receive recipient feedback immediately.<br>Only a few participants didn't say anything during the recording because they felt embarrassed. Still, most of them reported feeling more relaxed with the idea after receiving the audio from their pairs.<br>The sounds served to increase the sense of awareness of each other and most of the participants listened to the recorded audios carefully, sharing the activity only	Participants, divided into pairs, exchanged recorded audio messages during a solo meal and then listened to the audio message of their partners at a second solo meal.

					with the meal.<br>Some of the participants directly expressed their emotions during the audios. On other occasions, recipients reported being able to understand each other's emotions and mood through the pair's rhythm, behavior and passive expressions.<br>Yet, the heightened sense of physicality also contributed to the type and degree of intimacy felt among participants. As an illustration, the sound of chewing certain foods made the pair feel like sharing them.	

Binge drinking and obesity-related eating: the moderating roles of the eating broadcast viewing experience among korean adults	2021	Jiye Kim, Saegyeol Choi, Hyekyeong Kim e Soontae An	Quantitative	To identify the interaction between consumption of binge eating transmissions and obesity-associated eating behaviors among Korean students. Also, to find out if obesity-associated eating behavior was affected by interaction with compulsive eating and drinking	Association between the frequency of binge drinking, obesity-related eating behaviors and the experience of watching food streams: Binge drinking did not vary according to the experience of watching food streams. Also, people who watched more food broadcasts on TV and the internet scored more points on the topic of emotional eating and external eating (eating in response to external stimuli) than those who only watched food broadcast on TV.<br><br>Moderating effect of binge-watching experiences on the relationship between binge drinking and obesity-related eating behaviors: The interaction between binge drinking and binge drinking had a significant effect on binge eating behaviors, but not about food restriction and emotional eating. In a subgroup analysis, it was observed that the moderation effect was only observed in women.	Mukbang - a person eating large amounts of food while streaming.
Compensatory Usage of the Internet—The Case of Mukbang Watching on	2021	Kagan Kircaburun, Sabah Balta, Emrah	Quantitative	Investigate the problematic use of Youtube and its relationship with the problematic consumption of mukbang	Problematic mukbang consumption was associated with loneliness and problematic use of Youtube	Typical interaction in mukbang communities (mukbang videos or videos + chat)

YouTube		Emirtekin, Sule Betul Tosuntas, Zsolt Demetrovics e Mark D. Griffiths				
Dietary life and mukbang- and cookbang-watching status of university students majoring in food and nutrition before and after COVID-19 outbreak	2021	Hyunjoo Kang, Sowon Yun e Hongmie Lee	Quantitative	With the increase in time spent at home due to the extension of online classes, the study aimed to determine how the COVID-19 pandemic impacted the consumption pattern of mukbang and cookbang and the food consumption of university students.	Cookbang and mukbang consumption patterns<br>mukbang consumption in 2019:<br>occasional: 45.8%<br>frequent: 43.7%<br>mukbang consumption in 2020:<br>occasional: 42.5%<br>frequent: 35.3%<br><br>Cookbang consumption in 2019:<br>occasional: 43.7%<br>frequent: 19.0%<br>Cookbang consumption in 2020:<br>occasional: 45.5%<br>frequent: 24.6%<br><br>The proportion of participants who watched mukbang 5-7 days/week doubled and the proportion of cookbang participants tripled.<br>YouTube was the most used platform for video consumption.<br><br>Perception of dietary changes attributed to mukbang and cookbang consumption<br><br>Report of worsening diet in 2019 associated with	Consumption of videos of people eating or preparing food on video platforms

					mukbang: 18.1%<br>in 2020: 14.6%<br>Report of improved diet in 2019 associated with mukbang: 3.4%<br>2020: 4.6%<br><br>Report of improved diet in 2019 associated with the cookbang:<br>27.0%<br>2020: 35.9%<br>Report of worsening diet in 2019 associated with cookbang:<br>2.2%<br>2020: 1.7%<br><br>"Dietary life" related to mukbang and cookbang consumption<br>Cookbang consumers cooked more in both years and started eating alone more often in 2020.<br>In 2019: Mukbang consumers often rated themselves more negatively than non-consumers, considering consumption of processed foods, takeaways and sweets. However, this difference could not be observed in 2020.<br>In 2019, cookbang consumers rated their diet negatively in terms of consumption of caffeinated beverages (more than 3 cups/day), while they fared better in terms of milk and dairy consumption, both in terms of consumption. 2019 and in 2020.	
Exploring Possibilities to Improve Distant Dining Experience for	2021	Gursehaj Singh Arora	Mixed methods	The research work to provide a solution for the time and space gap issue between different target groups and their loved ones does exist	Augmented reality did provide a better sense of physical presence in the same room than video call and helped establish an improved sense of connectedness among diners using the shared elements and the augmented reality experience proved to be more	Eating with a remote commensal using videoconference technologies

Young Expats with their Loved Ones over Time and Space Gap Issues				and acquires different technologies to achieve the goal.	acceptable than a regular video call distant dining experience among young expats	
Effects of Dietary Similarity on Conversational and Eating Behaviors in Online Commensality	2021	Jui-Ying Wang, Sho Kubata, Jianpeng Zhanghe, Tomoo Inoue	Quantitative	To investigate the impact of meals made with the same foods through remote forms of co-eating, in order to examine how to utilize the social functions of commensality, even remotely, and provide design recommendations to support the practice of commensality	Conversation:<br>The proportion of speeches was the same between the situation with the same food and with different foods. The situations of silence were lower in the situation with the same food, which suggests that there was more dialogue in this condition.<br><br>Food intake:<br>Participants spent a little more time chewing and picking up food in the same-plate condition, although the authors stress that there were no statistically significant differences when compared to the different-food condition.<br><br>Looking:<br>Participants in the same-food condition spent more time looking at other things or looking away from the screen more often.	Participants had remote meals mediated by videoconferencing tool
El comer en el siglo XXI: Una aproximación a las sensibilidades en torno a la comida en Instagram [Eating in the 21st century:	2021	Constanza Faracce Macia, María Victoria Mairano	Qualitative	To reflect on the characteristics that the food practices of the 21st century have acquired from the reconfiguration of the sensitivity policies of "Society 4.0"	Food for enjoyment and conscious eating: between pleasure and body care:<br>The #foodporn, #yummy, #eatclean and #healthyfood, in general, represents dishes and are accompanied by captions with recipes and recommendations and advice to improve their preparations. The photos try to be professional (technical) and represent food as the protagonist.<br>Food, in this context, is both a digital object for consumption and a way to evoke emotions associated	Formation of communities in social networks and their dialogues with public food policies

An approach to the sensitivities around food on Instagram]					with people's desires.<br>In #foodporn and #yummy, processed foods are represented, associated with "pornography", exuberance and pleasure from consumption.<br>In contrast, #eatclean and #healthyfood are represented by fresh foods, with balanced amounts and nutritionally thought. They are crossed by gender and age structures and their repercussions on the pleasure of eating, moving and shaping the body.<br><br>Food practices in diners and community cooks: between the need and the possible<br>In the #ollassolidarias, #ollapopular, #merenderos, #meriendasolidaria, images of the daily activities of diners in situations of social vulnerability and cooks "toasting" to "free food assistance". (↑ COVID-19).<br>The food images that star in these # show large amounts of items that make up packaged basic baskets.<br>From a sociological perspective, public policies — particularly policies to promote healthy eating — shape bodies and emotions. Thus, taking into account the increase in food and nutritional insecurity, such public policies cross bodies notably marked by obesity, overweight and hidden hunger.<br>Finally, the aesthetics of food, in this context, is linked to both the need and the moral and affective dimensions that cross the practice of eating.	
Guardian of the Snacks: Toward designing a companion for mindful	2021	Rohit Ashok Khot, Daniel Prohasky	Qualitative	Explore the role of a multimodal company to interact in a solitary meal and still nurture healthy habits in snacks.	Participants learnt from their behavior and reduced the amount of their preferred snack to fill in the bowls. Reducing the amount worked as participants were still able to satisfy the sweet cravings, while being mindful of the sugar intake.<br>Interestingly, the burping sound, which was meant to	snacking with a robotic pet

snacking					signal the end of the snacking, actually served as an indication of “it’s close to dinner time.” So, when it was triggered, participants turned off the system and started preparing for their dinner.	
Reflection: Snatched Commensality: To eat or not to eat together in times of Covid-19 in France	2021	Estelle Fourat, Tristan Fournier, Olivier Lepiller	Qualitative	To explore how the lockdown affected rules and forms of commensality in France	With regard to commensality, new ways were identified in order to deal with this circumstance: 1) Commensality with covid prevention care - even in the lockdown, people gathered, but took care of hygiene. 2) Remote Commensality: meet friends virtually to drink and 3) "Commensality Ersatz (substitute)": share food photos, prepared recipes via whatsapp.	Commensality with neighbors and family with and without preventive care against covid. Remote commensality, forced commensality, overdose of commensality
The Playful Potential of Shared Mealtime: A Speculative Catalog of Playful Technologies for Day-to-day Social Eating Experiences	2021	Ferran Altarriba Bertran, Alexandra Pometko, Usa Muskan Gupta	Qualitative	To explore how play can be a lens through which we can rethink food technology innovation in ways that transcend productivity and embrace (and value) the social, cultural and emotional functions of mealtime.	The study presents recommendations to encourage designers to develop strategies that use the potential of technologies to facilitate interaction during meals: (1) using technology to deliver fun facts during the meal can stimulate social eating experiences; (2) in social mealtime experiences, it might be better to provide diners with agency over the unfolding of events rather than letting the technology scaffold the experience; (3) providing technology with some kind of personality, as opposed to making it appear neutral, might help people to see it as a fun agent that is socially aware and can be interacted with; (4) technology should embrace the messy, embodied, and hands-on nature of eating, leveraging it to the benefit of a rich mealtime experience; and (5) designers should consider how the idiosyncrasies of each context might impact how people experience a playful and social meal, since food, play, and social interactions are remarkably culturally loaded. We hope that these learnings help designers to attune the interaction mechanisms and underlying design qualities of their food-tech designs, in ways that the experiences afforded by the final	Interactions through games during meals and with the use of technologies

					outcomes are playful and socially stimulating.	
The use of ICT devices as part of the solo eating experience	2021	Mailin Lemke, Hendrik N.J. Schifferstein	Qualitative	To investigate how solo diners use digital devices as part of their daily eating experience	"The lived experience of solo diners and the role that ICT devices play as part of the eating experience is complex. Solo diners seem to use a number of different devices, depending on the context, purpose of use, mood and perception of convenience. The various applications that the ICT device can access can help to improve the eating experience, miti- gate potential negative emotions arising out of the context, perform secondary tasks due to the 'free' time while eating a meal and maintain one's social identity and social network. However, the devices also create distraction, which affects food perception, often leading to more and faster eating. Nonetheless, the devices can also contribute to healthy eating behavior, by providing food education, supporting mindful eating and by facilitating access to vulnerable populations, such as children and teenagers."	Eating alone with the use of technological devices
Virtual wine tastings—How to 'zoom up' the stage of communal experience	2021	Stefanie Paluch & Thomas Wittkop	Qualitative	To analyze the perception of the consumption experience in virtual wine tastings	The virtual wine tasting requires more preparation from the enthusiasts (look for a winery, for a date, type of event...). The winemakers/sommelier (hosts) are the central element of the events. The participants missed interpersonal elements, which was associated with a de-emotionalization of the experience and enhanced the focus on the knowledge. The elimination of spatial restrictions facilitated access to new customers. The participants noticed that they had similar interests with others, exchanged memories for building relationships and, by interacting with the group, they expanded their personal identities.	The tasting event itself, in which a script is followed and everyone together (even if separately), taste the wine mediated by computer, cell phone through videoconferencing software (zoom)
Waste on the Tip of the	2021	Lina Qu	Opinion article	To investigate online eating (chibo) broadcasts in the	The article presents a historical overview of mukbang in China and problematizes the phenomenon in the	Chibo

Tongue: Social Eating Livestreams (Chibo) in the Age of Chinese Affluence				context of China's anti-food waste campaign.	cultural and political context of the country, relating that the "Chinese dream" propagates abundance, and with that Chibo (the name given to characterize the Chinese mukbang) may be contributing for food waste.	
Watching a remote-video confederate eating facilitates perceived taste and consumption of food	2021	Nobuyuki Kawaia, Zhuogen Guoa, Ryzaburo Nakata	Qualitative	To investigate whether watching a silent video of others eating amplified preferences for food and food intake compared to watching silent videos of others' non-food related behavior or objects.	Participants noticed that popcorn tasted better only when they watched videos of other people eating it. Watching other people eat induced participants to eat more than watching the other two videos. This study indicates that remote video confederates increase not only food intake but also food taste perception.	"Remote-video Confederate": eating alone while watching someone else eat via video and eating alone while watching video without eating.
Digital Ethnography of Living Food: A Methodological Approach to Online and Offline Fieldwork	2021	Helena Varella; Monica Machado	Qualitative	To present the methodological design of digital and visual ethnography built to understand the digital sociability and subjectivity of live food diners in the Terrapia community.	The forms of digital sociability enhanced the aesthetics of live food through the sharing of photos of different food-style preparations, such as the representation of drawings on plates in the form of hearts, mandalas, butterflies and flowers. With that follows the idea of eating with the eyes.	Eating with eyes for those who see the published food photos and eating together in person in the community project.
FoodChattAR: Exploring the Design Space of Edible Virtual Agents for Human-Food Interaction	2021	Philip Weber, Kevin Krings, Julia Nießner, Sabrina Brodesser, and Thomas	Qualitative	To understand human-to-food interactive communication	Participants devoted considerable time to interact with the prototype and their food generally got cold. Some participants were so concentrated on the prototype that they slept pulling their fries on sauce, contributing to the atestment of the immersive — and distracting — power of it. The communication with the prototype was described as unnatural, but still led to amusement. The participants described the food as an entertainer.	Eating interacting with food in augmented reality

		Ludwig				
